# Green Synthesis of Fluorescent Carbon Dots and AI-Driven New Paradigms: A Comprehensive Review

**DOI:** 10.3390/bios16070356

**Published:** 2026-06-26

**Authors:** Qian Wang, Huiyao Liang, Xiaofeng Chang, Huili He, Rong Li, Jian Mao, Weiwei Han, Ying Tang, Yongfei Li, Maogang Li, Qunzheng Zhang

**Affiliations:** 1 Shaanxi University Engineering Research Center of Oil and Gas Field Chemistry, Xi’an Shiyou University, Xi’an 710065, China; 2 Shaanxi Province Key Laboratory of Environmental Pollution Control and Reservoir Protection Technology of Oilfields, Xi’an Shiyou University, Xi’an 710065, China; 3 Shaanxi Engineering Research Center of Green Low-Carbon Energy Materials and Processes, Xi’an Shiyou University, Xi’an 710065, China; 4 Xi’an Key Laboratory of Low-Carbon Utilization for High-Carbon Resources, Xi’an Shiyou University, Xi’an 710065, China; 5 CCDC Changqing General Drilling Company, Xi’an 710021, China; 6 Karamay Sanda High-Tech Co., Ltd., Karamay 834000, China; 7 State Key Laboratory of Heavy Oil Processing and Center for Bioengineering and Biotechnology, China University of Petroleum (East China), Qingdao 266580, China

**Keywords:** carbon dots, green synthesis, machine learning, sensing, bioimaging, catalysis

## Abstract

Carbon dots (CDs) have been widely employed in diverse fields by virtue of their excellent water solubility, low toxicity, high fluorescence stability, and favorable biocompatibility. Nevertheless, traditional preparation methods for CDs generally suffer from drawbacks that run counter to the concept of green chemistry. This review comprehensively summarizes the green synthesis technologies, machine learning (ML)-assisted synthesis strategies, and diversified application fields of fluorescent CDs. Specifically, it discusses the characteristics of synthetic organic molecular/polymeric materials and natural sources (e.g., plants and fruit peels, etc.) and elaborates on the top-down and bottom-up green synthesis methods, analyzing their advantages. It also focuses on ML’s core role in precisely regulating CD emission wavelengths, enhancing and predicting fluorescence quantum yields to optimize synthesis processes. Additionally, this review highlights the representative biological applications of CDs, including biosensing and biomedicine (e.g., bioimaging, drug delivery, and photodynamic therapy), while briefly covering their applications in other fields. Finally, the review points out current challenges in green synthesis, ML-assisted applications and industrial translation, and puts forward future research directions, aiming to promote the greenization, intellectualization and large-scale development of CDs.

## 1. Introduction

Carbon dots (CDs) are a class of zero-dimensional carbon-based nanomaterials that have evolved into non-toxic alternatives to traditional semiconductor quantum dots due to their high photoluminescence, stability, tunable emission wavelengths, and diverse applications, and have recently attracted extensive attention across various scientific fields. Following the serendipitous discovery of fluorescent carbon nanoparticles by Xu et al. [1] in 2004, Sun et al. [2] first used laser ablation of carbon targets in 2006 and formally named the surface-passivated highly fluorescent nanoparticles CDs, ushering in the rapid development of this emerging research field. CDs are spherical or quasi-spherical particles with sizes ranging from several nanometers to tens of nanometers [3]; CDs are renowned for their bright and tunable photoluminescence (PL), stability, and biocompatibility [4], making them the most promising candidates for various applications.

CDs typically consist of amorphous or nanocrystalline cores with graphitic structures composed of sp^2^-hybridized carbon atoms [5]. The graphitic (sp^2^) domains in CDs enhance electrical conductivity, photostability, and electron mobility due to their crystalline structure, and provide stability under light irradiation. The sp^3^ domains in CDs offer sites for extensive surface functionalization, thereby improving chemical reactivity, solubility, and biocompatibility. This amorphous structure endows CDs with stability in various environments, making CDs containing sp^3^ domains ideal materials for drug delivery, biosensing, and environmental applications—fields that demand precisely tunable surface properties and biocompatibility. The surfaces of CDs possess functional groups such as hydroxyl, carboxyl, and amino groups, which enhance their hydrophilicity and water solubility, rendering them particularly suitable for biomedical [6] and environmental applications [7]. These functional groups also enable facile modification, thereby improving targeted drug delivery, biocompatibility, and pollutant-sensing capabilities. CDs are regarded as active photocatalysts when doped with various metal ions, heteroatoms, and different functional groups. They can act as electron acceptors and donors [8], with extensive applications in chemistry and biology, including bioimaging [9], gene transfer [10], sensing [11], and catalysis [12].

In the early stage of the development of fluorescent CDs, the preparation methods mostly relied on physical methods, such as arc discharge [1] and laser ablation [2], or chemical methods, including strong acid oxidation and chemical synthesis. These traditional preparation methods are often plagued by issues such as harsh reaction conditions, high energy consumption, utilization of toxic and harmful reagents, and low yield, which not only limit the large-scale production of CDs but also contradict the concepts advocated by green chemistry, such as “atom economy” and “reduction in the use of harmful substances”.

With the growing emphasis on environmental protection and sustainable development, the development of green, efficient, and low-cost fluorescent CDs has emerged as a key research direction in current scientific studies. Numerous studies have focused on the properties and applications of CDs, yet the importance of carbon sources is often overlooked [13]. Green CDs synthesized using renewable natural resources such as vegetables, fruits, and agricultural wastes as carbon sources have emerged as a global research hotspot, owing to their unique advantages, including low cost, easy accessibility, high stability, simple synthesis procedures, environmental friendliness, non-toxicity, and the ability to realize the upcycling of low-value biomass wastes. Green preparation methods aim to achieve the controllable preparation of fluorescent CDs by utilizing renewable natural biomass resources or non-toxic and harmless chemical raw materials, through mild reaction conditions and simple operation processes. This approach thereby reduces environmental impacts and promotes the industrial application process of carbon dot materials. However, most of their emission wavelengths fall within the visible and near-infrared spectral regions below 1000 nm (NIR-I), and CDs exhibit low photoluminescence efficiency in the long-wavelength range, which limits their applications in photonics, optical fiber communication, and biology [14]. Therefore, to prepare multicolor CDs with excellent photoluminescence properties, researchers have conducted various studies on the synthesis, surface functionalization and modification, and optimized purification methods of CDs. Currently, trial-and-error methods based on manual experience remain the primary approach for preparing CDs. Nevertheless, such traditional methods inevitably involve subjectivity, randomness, and contingency, making it difficult to purposefully synthesize CDs with ideal photoluminescence properties. Synthetic factors affecting the photoluminescence of CDs include reaction precursors and their dosages, reaction time, reaction temperature, and solvent type [15]; appropriate adjustment and modification of these influencing factors can enhance the photoluminescence performance of CDs.

The swift development of artificial intelligence (AI) is profoundly transforming livelihood service models, with inclusive access to technology being realized through the widespread diffusion of technology.AI is defined as computer algorithms that can independently learn from input data without human intervention, and it has garnered remarkable accomplishments in diverse fields such as autonomous driving, speech recognition, and medical image processing. In the field of fluorescent CDs, AI can, by virtue of machine learning (ML) algorithms and based on multi-dimensional large-scale datasets including precursor types, reaction temperature, reaction duration, and solvent ratio, establish relevant models to rapidly screen optimal synthetic process parameter combinations. It can also accurately predict the regulatory effects of modification strategies (e.g., precursor doping and surface modification) on the emission wavelength and fluorescence quantum yield of CDs, thereby enabling the targeted design and controllable fabrication of fluorescent CDs with multicolor luminescence, long-wavelength emission, and high stability. With the continuous development of portable handheld sensors, AI is increasingly integrated with them to conveniently analyze and intelligently process data at a speed unmatched by humans [16]. To date, most optical and miniaturized sensors are used for target detection, demonstrating enormous potential in applications such as on-site monitoring and rapid point-of-care analysis. Among them, integrated handheld sensors, integrated smartphones, test strips/biochips, and customized 3D-printed accessories have become ideal platforms for chemical and biological quantitative analysis. For example, smartphone sensing platforms equipped with fluorescent carbon dot test strips analyze the changes in fluorescent signals of biomarkers (e.g., methylation [17] and uric acid [18]) in urine/saliva via Convolutional Neural Networks (CNNs), featuring high detection accuracy and enabling patients with chronic diseases to complete some routine tests without leaving their homes.

This review summarizes the research progress concerning carbon source types, synthesis strategies, and machine learning-assisted development of CDs, covering both top-down and bottom-up synthetic routes, with a particular focus on the natural carbon source systems of CDs. Compared with chemically synthesized CDs, natural-source CDs can be fabricated from a wide range of low-cost renewable materials, feature prominent performance advantages, and have garnered extensive attention in the research community. More notably, machine learning-assisted CD preparation permeates the entire R&D process of fluorescent CDs, realizing the intelligent optimization of synthetic procedures as well as the targeted regulation and precise prediction of optical properties, thereby effectively overcoming critical bottlenecks in traditional research such as high trial-and-error costs and difficulties in performance prediction. Smartphone-based sensing platforms enable on-site high-sensitivity detection, driving the translation of CDs from laboratory research toward green and accessible civilian applications. Additionally, this review systematically elaborates the application advances of CDs in multiple fields, including fluorescent probes, biomedicine, corrosion inhibition and protection, and catalysis.

## 2. Sources of CDs

### 2.1. Synthetic Organic Molecular/Polymeric Materials

The CDs derived from synthetic sources use artificially synthesized chemicals or polymer materials as precursors. The precursor ratio and reaction conditions are regulated, and precise control over particle size, surface functional groups, and fluorescent properties can thus be achieved. To date, several chemical precursors, including thiourea [19], dopamine [20], triammonium citrate [21], zinc oxide [22], boric acid [23], and urea [24], have been employed for the synthesis of CDs (Table 1).

#### 2.1.1. Organic Molecules

**Figure 1:**
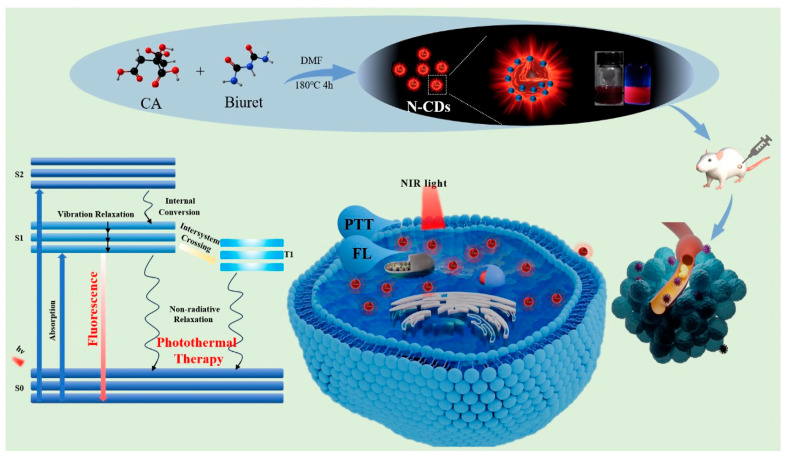
Schematic illustration of the preparation and tumor ablation of N-CDs [30].

In recent years, small organic molecules have been used as raw materials to regulate properties via doping (N, S, P, etc.) or compositing (e.g., with metal nanoparticles). For instance, Shen et al. [25] prepared full-color fluorescent CDs via the solvothermal method, using citric acid and urea as precursors, and N,N-diethylformamide as both solvent and eluent. Xu et al. [26] developed a rapid hydrothermal carbonization method using glucose as the carbon source, with a synthesis time as short as 0.5 min—far more efficient than conventional hydrothermal carbonization approaches. El-Said et al. [27] modified CDs synthesized via a green synthesis route with gold nanoparticles (Au NPs) of different concentrations; the resulting CDs/Au NP nanocomposites were applied as optical nanosensors for neurotransmitters. Aromatic compounds can be used to synthesize CDs with long-wavelength emission. For example, Dong et al. [28] improved the quantum yield (QY) of o-phenylenediamine-based red-emissive carbon quantum dots (R-CQDs) by introducing ethylenediamine, preparing red-emissive carbon quantum dots with a QY of 32.65%. Li et al. [29] synthesized doped CDs via a microwave-assisted method, using o-phenylenediamine (OPD) and L-lysine (L-LYS) as raw materials, boric acid or phosphoric acid as different heteroatom dopants, and water as the solvent. Two types of doped CDs with distinct fluorescence colors under 365 nm ultraviolet (UV) light were ultimately obtained: B-doped CDs exhibited orange fluorescence, while P-doped CDs showed red fluorescence. This indicates that different heteroatom dopants can alter the fluorescence color and emission wavelength even when the same precursors are employed. Shi et al. [30] synthesized nitrogen-doped carbon dots (N-CDs) through a facile one-pot hydrothermal strategy with citric acid and urea as precursors. Tuning the sp^2^-conjugated domains, surface oxidation level, and nitrogen doping enables bandgap engineering and fluorescence red-shift. The as-prepared N-CDs display the strongest fluorescence emission at 660 nm along with superior photothermal stability and dual near-infrared light absorption, whose photothermal conversion efficiencies reach 31.25% and 27.12% upon 808 nm and 1060 nm laser irradiation, respectively. These N-CDs hold great promise for applications in fluorescent bioimaging, antibacterial treatment, and integrated tumor theranostics (Figure 1).

#### 2.1.2. Polymers and Industrial Raw Materials

Polymers and fossil fuel derivatives can also serve as sources of CDs. Polymers can form CDs with surface-enriched functional groups through cross-linking and carbonization, making them suitable for sensor construction. Li et al. [31] heated a mixture of selenocysteamine hydrochloride and m-phenylenediamine at 180 °C for 10 h in a high-pressure reactor, then took the supernatant and added polyacrylic acid, followed by dialysis purification, to obtain polyacrylic acid-coated Se-doped CDs with high biocompatibility. Şen et al. [32] synthesized fluorescent polyethyleneimine-capped carbon quantum dot probes via microwave-assisted technology using polyethyleneimine and citric acid as precursors, which were applied for the detection of trinitrotoluene (TNT). Fossil fuel derivatives are typically converted into CDs via chemical cleavage or electrochemical oxidation and are commonly used in the field of photocatalysis. Molaei et al. [33] synthesized N-doped carbon quantum dots (N-CQDs) by subjecting carbon black to rigorous oxidation conditions followed by N-doping with hexamethylenetetramine and polyethyleneimine; these N-CQDs can be internalized into cells and used for fluorescent cell imaging.

**Table 1:** Synthesis of CDs from synthetic sources.

Carbon Source	Heteroatom Doping	Synthesis Methods	Fluorescence	Application	QY (%)	Ref
Citric acid	Tris(hydroxymethyl) aminomethane	Hydrothermal	Blue	Detection and anti-countereiting of ciprofloxacin	53	[34]
Citric acid	Thiourea	Hydrothermal	Blue	Detection of tartrazine	-	[35]
Glucose	O-phenylenediamine	Hydrothermal	Yellow	Fluorescent probes for rutin detection	-	[36]
Starch	Urea	Microwave -assisted hydrothermal	Blue	Bioimaging	7.6	[37]
3-aminobenzoic acid	Benzenesulfonamide	Hydrothermal	Yellow	Drug delivery	-	[38]
Citric acid	L-Asparagine	Hydrothermal	Blue	Fluorescent probes for bilirubin	36	[39]
O-phenylenediamine	Ethylenediamine	Hydrothermal	Green	Detection of Hg^2+^	23.1	[40]
O-phenylenediamine	Phytic acid	Hydrothermal	Red	Detection of 2-methylimidazole	-	[41]
Citric acid	4,4′-Dipyridyl	Hydrothermal	Green	Fluorescent probe for pH	4.03	[42]
P-aminophenol	Ethylenediamine	Hydrothermal	Yellow	Detection of Al^3+^	10.74	[43]
O-phthalaldehyde	Polyethyleneimine	Hydrothermal	Orange	Detection of metal ions	38.64 (H^+^)	[44]
O-phenylenediamine	Semicarbazide	Hydrothermal	Orange	Warm white LED and fluorescent ink	23	[45]
1-amino-2 -naphthol -4-sulfonic acid	Ethylenediamine	Microwave- assisted solvothermal	Green	Fluorescent ink	33.8	[46]
Quercetin	O-phenylenediamine	Hydrothermal	Yellow	Detection of Ag^+^ and glutathione	11.3	[47]
Disodium ethylenediaminetetraacetate	Boric acid	Hydrothermal	Blue	Information encryption and anti-counterfeiting	10.28	[48]
Aniline	Urea, ethylenediamine	Hydrothermal	Blue	Detection of CO^2+^	22.67	[49]
Citric acid	Urea	Hydrothermal	Red, blue, green	LED and fingerprint detection	5.87 13.18 15.89	[50]
Ethylenediaminetetraacetic acid	Diammonium hydrogen phosphate	Microwave-assisted synthesis	Blue	Detection of rutin	63.8	[51]
Citric acid	Urea	Microwave -assisted synthesis	Blue	Detection of Hg^2+^ in wastewater	60.51	[52]
2,5-diaminobenzenesulfonic acid, 2,4-dihydroxybenzoic acid	Cuprous chloride	Hydrothermal	Yellow	Detection of urease and proteus	10.41	[53]
Carboxymethyl cellulose	L-cysteine	Hydrothermal	Blue	Detection of tetracyclines and Al^3+^	44.48	[54]
Citric acid	Dopamine	Hydrothermal	Blue	Detection of Fe^3+^ and LED devices	15.7	[55]
1-(2-pyridylazo) -2-naphthol	Terbium nitrate hexahydrate	Hydrothermal	Blue	Detection of protein cytochrome C	-	[56]
Chitosan	Ethylenediaminetetraacetic acid	Hydrothermal	Blue	Fe^3+^ and AA detection with encryption	4.77	[57]
Sucrose	Urea	Microwave-assisted	Blue	Determination of torsemide and plasma samples	0.57	[58]
Lactose	4-amino-3-hydroxy-1-naphthalenesulfonic acid	Hydrothermal	Cyan -green	Detection of Ga^3+^ and riboflavin	16.7	[59]
Chrysoidine G	Potassium ferrocyanide	Electrolytic	Green	Detection of Cr^6+^	6.05	[60]
L-cysteine	Neutral red	Hydrothermal	Red, orange, green	Apigenin detection and adsorption application	14	[61]
Sucrose	L-cysteine/D-cysteineand rhodamine B	Hydrothermal	Pink	Visual fluorescent Ag^+^ sensing	19.46 18.74 6.33	[62]
L-lysine	Ethylenediamine	Hydrothermal	Blue	Temperature sensor and detection of tinidazole	-	[63]
L/D-glutathione	Porphyrin	Hydrothermal	Blue	Dual-ratiometric fluorescent sensor for arginine	-	[64]
O-phenylenediamine	Boric acid and Aluminum nitrate	Hydrothermal	Yellow	Detection of F^−^ in cells and zebrafish	34.68	[65]

### 2.2. Natural Sources

Natural carbon sources use carbon-rich biomass from nature as raw materials, encompassing plants, animals, and microorganisms (Table 2). Typically, CDs prepared from small organic molecules may be costly and potentially toxic. Therefore, methods utilizing green and readily available raw materials have been explored. “Green” CDs have attracted extensive attention from researchers due to their excellent water solubility, outstanding biocompatibility, and environmental friendliness, and can be fabricated from a variety of low-cost and renewable materials. However, natural biomass precursors feature complex compositions and significant batch-to-batch heterogeneity, and the synthesis process is governed by coupled multi-parameter effects. Consequently, the fluorescence regulation of CDs has long relied on empirical trial and error, with inherent drawbacks including difficult directional design and poor batch reproducibility. In recent years, ML has emerged as a novel data-driven research tool. Database-based model construction enables high-throughput screening of biomass precursors and intelligent optimization of synthesis processes, advancing CD fabrication from an empirically driven mode to rational and controllable preparation.

#### 2.2.1. Plant-Derived Sources

CDs are typically synthesized from various natural and food sources via microwave and hydrothermal methods. Fruits and vegetables are rich in carbohydrates, organic acids, and natural fluorophores, are abundant in nature, and can serve as carbon precursors for CD synthesis. CDs derived from plants are typically low-toxic and suitable for biomedical applications such as bioimaging and drug delivery. For instance, Wang et al. [66] prepared BP-N-CDs via the hydrothermal method using banana peel as the precursor and o-phenylenediamine as the dopant, realizing the detection of resveratrol in food through the inner filter effect (IFE). Tan et al. [67] synthesized CDs from waste pitaya peel as the carbon source via a one-pot hydrothermal method; these CDs exhibited low cytotoxicity, were easily internalized by cells, and were applied for intracellular Fe^3+^ imaging and sensing. Renuga et al. [68] synthesized highly fluorescent CDs from bitter melon via a simple hydrothermal method, which could be used as Fe^3+^ ion sensors and featured excellent low toxicity, enabling broad applications in the biological field. Agricultural or industrial by-products such as straw, rice husk, bagasse, and wood waste can also be used to prepare CDs, which usually contain heteroatoms (e.g., N, S) that enhance fluorescent properties. For example, Ma et al. [69] synthesized N-doped CDs (N-CDs) from biomass bagasse via the hydrothermal method with a photoluminescence quantum yield (PLQY) of 32.6%, and developed a method for detecting methyl parathion through alkaline hydrolysis. Plant extracts (e.g., tea leaves, coffee grounds, and aloe vera juice) are rich in polyphenols and flavonoids and can generally be used to fabricate multifunctional CDs via a one-step method. Zhu et al. [70] synthesized Fe-doped carbon quantum dots in an eco-friendly way from waste coffee grounds via the hydrothermal method; the fluorescence of these CDs could be quenched via IFE and then recovered upon the addition of ascorbic acid. Tsai et al. [71] prepared three types of fluorescent CDs with blue, green, and red emission from pine needle powder as the main carbon source via a one-pot microwave reaction, which were applicable for the detection of carcinoembryonic antigen (CEA) and tumor necrosis factor-α (TNF-α) biomarkers.

#### 2.2.2. Animals and Derived Natural Products

Compared with traditional CDs, biomass-derived CDs synthesized from renewable resources eliminate the additional steps of introducing external heteroatoms and functional groups, thus exhibiting advantages such as strong luminescence, small size, excellent photostability against blinking and photobleaching, low toxicity, high electrical conductivity, and superior biocompatibility and chemical stability. Le et al. [72] successfully synthesized amino-functionalized blue-green fluorescent CDs with high QY using chitosan as the carbon precursor via a simple one-step green carbonization method. Cardoso et al. [73] obtained CDs with a PLQY of 25.6% from egg white in a one-step synthesis, which was completed within 6 h without any purification. The preparation of CDs from natural products provides a low-cost and abundant source for doped carbon quantum dots. Zhang et al. [74] synthesized milk-derived carbon quantum dots with blue fluorescence via the hydrothermal method using milk as the carbon source. Kamble et al. [75] synthesized carbon quantum dots from human hair as the carbon source without any chemicals; the resulting carbon quantum dots exhibited strong blue light emission with a PLQY of 28% and could be applied in bioimaging.

#### 2.2.3. Microorganisms

Due to the low cost, low toxicity, availability, and abundance of microorganisms, they have attracted considerable interest as precursors for CD synthesis. Microbial cells are abundant sources of organic molecules such as carbohydrates, peptidoglycans, proteins, and peptides, and contain numerous heteroatoms (typically O, N, S, and P); thus, they can create functionalized surfaces for biomass-derived CDs without any additional modification (i.e., doping). In 2016, Hua et al. [76] first reported the successful synthesis of fluorescent CDs from bacteria via a one-step hydrothermal carbonization method using Staphylococcus aureus or Escherichia coli cells; these CDs possessed multiple functional groups, high negative surface charge, and appropriate size, enabling bacteria-derived CDs to distinguish between live and dead microorganisms. Subsequently, microbial cells such as Thermococcus [77] have also been used for CDs synthesis. As an alternative to microbial cells, various extracellular metabolites produced by microorganisms during fermentation (the so-called postbiotics) can serve as starting materials for CDs synthesis. Ghorbani et al. [78] synthesized multifunctional CDs from metabolites secreted by Saccharomyces cerevisiae via the hydrothermal method, which could be used as antibacterial/antioxidant additives and antibacterial packaging materials. Algal materials are rich in chlorophyll, proteins, polysaccharides, and unsaturated fatty acids, and can form multicomponent synergistic fluorescent centers after carbonization. Zhang et al. [79] synthesized Spirulina-derived CDs with red fluorescence from Spirulina as a bio-based raw material via the hydrothermal process; these CDs featured ultrasmall size, could be applied in cell imaging, and achieved effective renal clearance.

**Table 2:** Synthesis of CDs from natural carbon sources.

Carbon Source	Fluorescence	QYs (%)	Application	Ref.
Peanut shell	Green	17.1	Bioimaging	[80]
Barberry	Blue	12.82	Detection of Fe^3+^	[81]
Sichuan pepper	Blue, green, red	13.1, 11.2, 9.8	Anti-counterfeiting ink	[82]
Crown daisy	Green	14.5	Anti-counterfeiting ink	[83]
Bagasse	Blue	32.6	Detection of sulfur and phosphorus	[69]
Solanaceous plants	Blue	35	Electrocatalysis and bioimaging	[84]
Amaranth	Red	42.0	Determination of water content in organic solvents	[85]
Lantana flower	Blue	29.0	Detection of Cr^6+^ and cell imaging	[86]
Waste coffee grounds	Cyan	19.73	Fingerprint detection	[87]
Guava-fruit	Blue	26.12	Determination of risperidone in plasma and pharmaceutical dosage forms	[88]
Butternut squash peel	Blue	13.5	Antioxidants and antibacterial agents	[89]
Ginkgo leaves	Blue, green, red	13.07; 6.09; 6.72	Fluorescent ink	[90]
Green tea	Blue	21.6	Detection of Cu^2+^	[91]
Green onion	Blue	21.3	Detection of rifampicin	[92]
Shrimp shell	Green	33.2	Visual portable pH measurement platform	[93]
Waste egg carton pulp	Blue	48.3	Fe^3+^ sensor	[94]
Cassava pulp	Blue	65.2	Detection of hypochlorite and cell imaging	[95]
Bay leaves	Blue	25	Cell culture, bioimaging	[96]
Heena leaf powder	Red	40	Cell labeling agents and bioimaging probes	[97]
Inulin	Blue	28.6	Fluorescent probe for rapid/visual detection of 3-nitrotyrosine	[98]
Flowering plum	Blue	35	Trace detection of chlortetracycline	[99]
Syzygium cumini seeds	Blue	52.8	Selective recognition of Fe^3+^ and Cu^2+^	[100]
Waste papaya seeds	Blue	30.02	Detection of ciprofloxacin and bioimaging	[101]
Malabar spinach seeds juice	Red	23.9	Fluorescent fibers fabrication	[102]
Boerhaavia diffusa	Cyan-green	21.2	A fluorescence turnoff sensor for ferric ion detection in aqueous solution	[103]
Eucalyptus globulus leaves	Blue	60.7	/	[104]
Embryos of cumin seeds	Blue	10.60	Detection of rutin	[105]
Fish scales	Blue	31.71	Zebrafish imaging	[106]
Dregea volubilis fruit	Yellow	59	Detect glucose in urine and blood samples	[107]
Dried pomelo peel	Blue	28	Determination of tartrazine	[108]
Walnut green skin	Blue	32.5	Detection of Pb^2+^	[109]
Quercus infectoria	Yellow	39.96	Dual-switch fluorescent sensor for Fe^3+^ and cysteine	[110]
Ignited peel of flame-roasted eggplant	Blue	28.5	Detection of hydrogen peroxide	[111]
Peels of peach palm fruit	Blue	25.4	Detection of Hg^+^	[112]
Sweet potato	Blue	53	Detection of Fe^3+^ and tobramycin	[113]
Peanut shells	Blue	56	Detection of Ag^+^ and Ba^2+^	[114]

## 3. Green Methods for CD Synthesis

The preparation methods for CDs are generally categorized into two types: “top-down” and “bottom-up”. The “top-down” method involves downsizing large-sized carbon materials such as graphite, carbon nanotubes, and nanodiamonds into small-sized CDs via physical or chemical approaches. In contrast, the “bottom-up” method synthesizes the CDs from small organic molecules or oligomers (including citric acid [115], ascorbic acid [116], and glucose [117]) through a series of chemical reactions.

### 3.1. Top-Down

**Figure 2:**
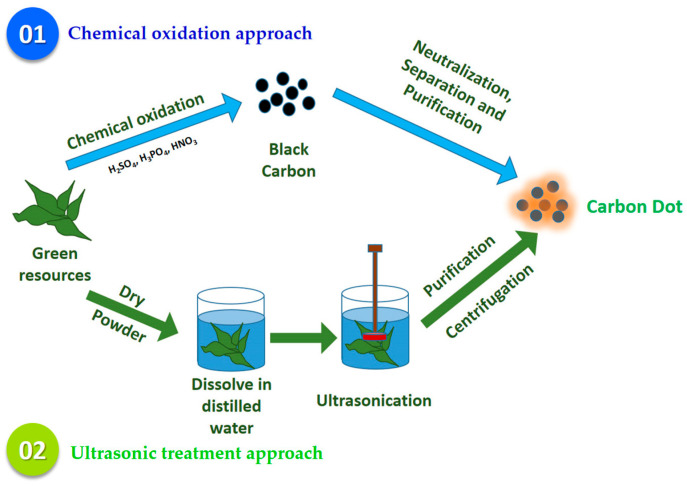
Top-down approaches in green synthesis of carbon dots [121].

As mentioned above, the “top-down” method refers to the decomposition of carbon materials into CDs. Common top-down methods include arc discharge, electrochemical method, chemical oxidation method, laser etching method, and mechanical ball milling method, among others. Compared with the “bottom-up” method, CDs synthesized via the top-down method exhibit several distinct advantages. One notable advantage of the top-down method is its ability to produce size-controllable CDs. Since CDs are prepared by decomposing bulk carbon precursors, the size of the resulting CDs can be regulated by adjusting the applied “cleavage force”. For instance, Tian et al. [118] proposed that electrochemical exfoliation using different electrolytes can yield CDs with varying sizes. Therefore, carbon precursors with a single component and well-defined structure (e.g., graphite, graphene) are mostly used to produce CDs via the top-down method. Most importantly, CDs prepared from bulk carbon materials through the top-down method typically possess well-defined surface functional groups, which are more amenable to further modification [119]. Thus, various functional groups, drug molecules, fluorophores, and even antibodies [120] can be grafted onto the surface of top-down-derived CDs, endowing them with desired properties and functions. The chemical oxidation method and ultrasonic treatment method are briefly introduced below (Figure 2).

#### 3.1.1. Chemical Oxidation Etching of Carbon Materials

Chemical oxidation is one of the most widely used top-down synthetic approaches for the fabrication of CDs. Fundamentally, the top-down strategy involves cutting and exfoliating bulk carbon precursors into nanoscale CDs via physical or chemical means. Conventional chemical oxidation methods employ strong oxidizing agents such as concentrated sulfuric acid, concentrated nitric acid, and potassium permanganate. Typically, CD precursors are mixed with these oxidizing agents, and under controlled temperature and reaction duration, the strong oxidizing acids carbonize organic moieties into carbonaceous materials, ultimately yielding CDs [122]. For instance, González-González et al. [123] used activated and non-activated pyrolytic carbon blacks derived from the pyrolysis of waste tires as precursors, performed chemical oxidation treatment with nitric acid, and prepared CDs with distinct PL properties in combination with activators. This method features simple operation and low raw material cost, and the resulting CDs possess ultrasmall particle sizes. However, this process necessitates the use of highly oxidizing and corrosive chemical reagents, posing inherent safety risks during preparation and violating the core principles of green chemistry. To address the inherent drawbacks of conventional chemical oxidation methods, researchers have developed a milder hydroxyl radical-based method by optimizing the core principle of oxidative scission. This approach belongs to the top-down category of CDs synthesis and, more specifically, represents an important branch and novel variant of chemical oxidation methods. Limosani et al. [124] synthesized carbon-based nanomaterials via the hydroxyl radical method, where CDs were obtained through hydroxyl radical-induced ring-opening of fullerenes using hydrogen peroxide, and applied them as fluorescent probes for metal ion detection and nuclear imaging. Luo et al. [125] used loofah sponge-based activated carbon fibers as raw materials, added hydrogen peroxide for oxidation and ultrasonic treatment, then subjected the filtrate to centrifugation, filtration, and dialysis to ultimately prepare CDs, and investigated the fluorescence-quenching effect of Cr^6+^ ions on the CDs.

#### 3.1.2. Ultrasonic Fragmentation Method

Ultrasonic treatment is one of the “top-down” methods. It applies high-energy ultrasonic waves to decompose large carbon molecules into smaller CD particles [125]. This method has been recommended due to its advantages, including environmental friendliness, low cost, strong penetration, and uniform effect [126]. For example, Xu et al. [127] ultrasonically treated kiwi fruit juice with different additives such as ethanol, ethylenediamine, and acetone, successfully synthesizing nitrogen-doped M-CDs with fluorescence emissions including green, yellow-green, and pink. Sahu et al. [128] used citric acid and glycerol as precursors, ultrasonically treated a mixture of lemon juice, glycerol, and distilled water for 6 h, then removed impurities via dialysis to obtain blue fluorescent CDs. Zaib et al. [129] adopted a simple method to prepare CDs: they used Polyalthia longifolia leaves as the carbon source, dried and ground them into powder, dissolved the powder in distilled water, ultrasonically treated the mixture for 1 h, and then purified it to obtain CDs. Zhao et al. [130] used natural biomass as precursors to prepare multicolor biomass-based carbon nanodots (CNDs) via an ultrasonic-assisted method at room temperature; cavitation generated by ultrasound in the solution facilitated the polymerization of biomolecules into nanodots. The PLQYs of the corresponding CNDs were 11%, 12%, and 28%, respectively.

### 3.2. Bottom-Up

The bottom-up strategy for synthesizing CDs mainly includes hydrothermal/solvothermal methods, microwave synthesis, combustion, and pyrolysis technologies. In particular, the hydrothermal method is commonly used for preparing green CDs due to the accessibility of raw materials and low production cost. Compared with the top-down strategy, these bottom-up methods typically provide more controllable morphologies and allow faster synthesis and surface modification via one-step operations. The bottom-up approach for CD synthesis is highly recommended by many researchers, attributed to its simple procedures, low cost, environmental friendliness, feasible large-scale production, and precise and controllable design of initial molecules. Another advantage of the bottom-up strategy lies in the diversity of its raw materials. Not only chemical reagents such as citric acid and ascorbic acid can be utilized, but also various biomasses including potato [131], pitaya peel [67], green acorn [132], watermelon rind [133], and Paulownia fortunei flowers [134] are applicable for CDs synthesis.

#### 3.2.1. Hydrothermal

**Figure 3:**
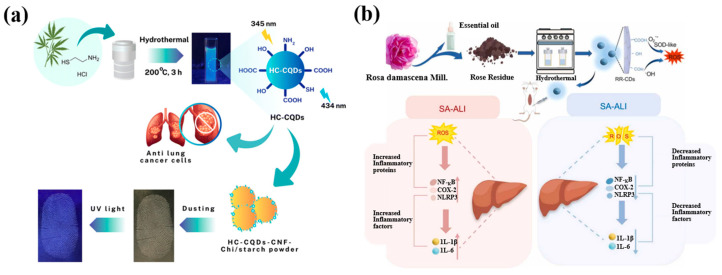
(**a**) Synthesis of HC-CQDs, which were utilized to detect LFPs and can possibly be used against lung cancer cells [140]; (**b**) green hydrothermal valorization of Rosa damascena Mill. floral waste into functional carbon dots [141].

Hydrothermal/solvothermal synthesis methods involve carbonization reactions of polymers or carbon sources in appropriate solvents under high-temperature and high-pressure conditions, via which CDs can be formed (Figure 3). These methods typically utilize organic acids, small organic molecules, polysaccharides, and some waste fruit peels as carbon sources. For instance, Raypah et al. [135] synthesized CDs via a hydrothermal method using a mixture of lemon and ginger juice as the carbon source, which exhibited a fluorescence QY of 27.7% and excitation-dependent emission, with maximum excitation and emission wavelengths of 400 nm and 480 nm, respectively. Cytotoxicity assays on HeLa and MCF-7 cell lines confirmed high cell viability (≥86%), indicating low toxicity and good biocompatibility—endowing them with great potential in bioimaging, drug delivery, and biosensing applications. Zheng et al. [136] successfully prepared blue fluorescent CDs with a QY of 42.96% via a one-step hydrothermal method using Pueraria lobata residue as the precursor and urea as the nitrogen source. Electron transfer between the CDs and hexavalent chromium in water could induce fluorescence quenching, enabling the CDs to serve as a fluorescent probe for Cr^6+^ detection with a limit of detection (LOD) of 0.078 μM and a limit of quantification (LOQ) of 0.26 μM. Notably, this fluorescent probe was also validated in various water matrices, achieving stable recovery rates ranging from 98.7% to 101.5%. Jing et al. [137] synthesized a novel green precursor-based nitrogen-doped carbon dot (N-CDs) probe via a simple solvothermal method using Portulaca oleracea as the precursor. The probe exhibited excellent sensitivity and selectivity towards MnO_4_^−^ ions, with a linear equation of y = 3.2039x + 30.6872, a correlation coefficient (R) of 0.9916, and a relative standard deviation (RSD) ranging from 1.85% to 8.75%. The linear detection range was 0.5 to 168 μM, with an LOD of 41.8 nM and an LOQ of 139.3 nM, providing an efficient and simple fluorescent nanoprobe method for monitoring MnO_4_^−^ ion residues in environmental water quality. Kolekar et al. [138] synthesized blue-emitting sulfur-doped carbon dots (S-CDs) using crude sugar as the carbon precursor via a one-step hydrothermal method, which showed selective and sensitive quenching responses towards Cr^6+^ and Fe^3+^ with LODs of 4.25 μg·mL^−1^ and 3.15 μg·mL^−1^, respectively. Zhang et al. [139] prepared biomass-derived carbon quantum dots (B-CDs) from bear bile powder via a one-step hydrothermal method. Notably, the B-CDs exhibited distinct concentration-dependent optical properties: they emitted green fluorescence at a high concentration (15 mg·mL^−1^) while emitting blue fluorescence at a low concentration (0.25 mg·mL^−1^). This unique feature enabled the B-CDs to act as a highly efficient probe for the detection of the antibiotic gatifloxacin with excellent performance, showing a wide linear detection range (0.01–100 μM) and a low LOD (8 nM).

#### 3.2.2. Microwave-Assisted

The microwave synthesis method has attracted increasing attention due to its advantages of being simple and saving time. Its basic principle is to utilize microwave radiation to drive the carbonization reaction of carbon sources and realize the production of CDs by controlling reaction conditions such as microwave radiation power and temperature. Microwave heating is characterized by rapidity and uniformity, which can make the reaction system reach the required temperature in a short time, thereby accelerating chemical reactions. The process of preparing carbon quantum dots via microwave method exhibits high controllability and easy optimization of experimental conditions. Compared with other methods, the microwave method eliminates many harsh experimental conditions and complex processes, thus greatly improving the preparation efficiency. In 2009, Zhu et al. [142]. proposed an economical microwave pyrolysis method for preparing fluorescent carbon nanoparticles with electrochemiluminescent properties. They used glucose and fructose as carbon sources, heated the mixture of polyethylene glycol (PEG) and sugars in a 500 W microwave oven for 2~10 min, and finally obtained carbon nanoparticles with excellent fluorescent properties. Yin et al. [143] synthesized a novel biomass carbon dot using Ipomoea aquatica and polyethylene glycol as raw materials via the microwave method, which was used as a fluorescent probe. Based on the IFE between the luminescent CDs and the harmful dye crystal violet (CV), the blue emission of the probe at 430 nm could be quenched by CV. The combination of the microwave synthesis method with different precursors and methods (e.g., hydrothermal method, pyrolysis method) has further expanded the application potential of CDs in fields such as biomedicine and optoelectronic devices. Gao et al. [144] achieved nitrogen–boron co-doping via a microwave-assisted hydrothermal method using nitrogen-rich biomass soybean meal as the raw material. With boric acid as the acidic proton source, the formation of B-C covalent bonds in the hydrothermal system was induced, and boron-doped carbon dots with a glassy structure were synthesized, enabling the application of biomass-derived CDs in information encryption. Liu et al. [145] synthesized magnetic fluorescent carbon dots (MF-CDs) with a size of approximately 2 nm via one-step microwave-assisted pyrolysis of citric acid and ethylenediamine. The superparamagnetism and fluorescent behavior endow MF-CDs with broad potential in multimodal cell imaging. This method is relatively simple and suitable for large-scale production, but it usually results in inhomogeneous sample size and requires complex separation processes. The raw materials used for preparing carbon quantum dots via the microwave method are generally relatively cheap and easily accessible. These raw materials are not only low-cost but also environmentally friendly, which is conducive to green production.

#### 3.2.3. Combustion

The combustion method requires simple raw materials, mainly including candle soot, natural gas soot, paraffin oil soot, and the like. In 2007, Liu et al. [146] first reported the preparation of CDs via the candle soot combustion method. They collected candle soot generated from incomplete combustion using aluminum foil, refluxed it in 5 mol·L^−1^ nitric acid solution, and finally obtained luminescent CDs with good dispersibility through centrifugation, neutralization, dialysis, and other processes. These CDs were subjected to electrophoretic separation in polyacrylamide gel, and nine types of CDs with a particle size of approximately 1 nm and gradually redshifted emission wavelengths under 315 nm excitation were obtained according to the migration rate in electrophoresis. Notably, the CDs prepared by this method exhibit a low QY. This is a time-consuming and costly synthesis method. In addition, it is difficult to separate templates from CDs, and the removal of templates may affect the purity, particle size, and luminescent properties of CDs. The yield of CDs produced by this method is relatively low, and the method is not suitable for large-scale production [147].

#### 3.2.4. Other Methods

In addition to the aforementioned methods, the preparation methods of CDs also include the pyrolysis method and reverse micelle method, etc. The pyrolysis method mainly uses organic compounds as carbon sources and forms CDs through the pyrolysis and carbonization of organic matter at high temperatures. This method offers a wide range of carbon sources for CD preparation; CDs with different properties can be obtained by controlling the degree of decomposition. It is simple, practical, and highly reproducible, and the resulting CDs exhibit high fluorescence QY. Wang et al. [148] synthesized phosphorus-doped carbon quantum dots (P-CQDs) via solid-phase pyrolysis, which were used as fluorescent probes for tetracycline detection. The reverse micelle method (RMM) is a chemical method for synthesizing fluorescent CDs by utilizing surfactants to form nanoscale microreactors (reverse micelles) in non-polar solvents. By regulating the size and reaction environment of reverse micelles, this method can effectively control the particle size, morphology, and fluorescence properties of CDs. A brief comparison of various synthesis methods of carbon dots is presented in Table 3.

## 4. ML-Assisted Synthesis of CDs

ML has revolutionized multiple fields by enabling computers to learn from data and make intelligent decisions [149]. ML involves training models on large datasets to identify patterns and make predictions [150]. ML models can easily handle tens or even hundreds of input features (e.g., precursor element content, synthesis temperature, reaction time, various spectral data, etc.). Traditional machine learning algorithms rely on manual feature engineering and mathematical algorithms [151,152]. In contrast, deep learning (DL), as a branch of ML [153], adopts a different approach: it uses artificial neural networks (ANNs) to automatically learn hierarchical feature representations of data. Deep neural networks consist of interconnected nodes that learn increasingly complex features, allowing the network to capture subtle patterns and relationships [152]. The development of ML has provided a new approach for researchers to overcome the challenges of optimizing CD synthesis [153,154,155]. The general workflow of ML consists of three major stages: data acquisition and preprocessing, model selection and training, as well as model evaluation and prediction [156]. With versatile multi-dimensional data-processing capabilities, ML standardizes model learning and performance assessment by dividing datasets into training, validation, and test sets, and employs cross-validation to mitigate random errors induced by data partitioning [157]. Aiming at common defects of raw data, including missing values, outliers, and redundant information, multiple optimization strategies such as data screening, dimensionality reduction, standardization, and feature encoding are adopted to improve data quality and unify data formats, thereby guaranteeing stable model operation [158]. Nevertheless, cross-validation can only reduce random errors from data segmentation, rather than thoroughly resolve issues like uneven sample distribution and inadequate sample representativeness. Consequently, well-trained models tend to generate biased predictions when applied to unseen samples and practical complex scenarios. Models should be selected pertinently according to research objectives and data categories, and mismatched algorithm selection will directly lead to invalid analytical results. Unsupervised learning merely explores inherent data structures passively and fails to achieve targeted prediction, while supervised learning relies heavily on fully labeled datasets, leaving unlabeled samples unavailable for prediction [156]. Each learning algorithm possesses inherent application restrictions. The final accuracy and generalization performance of models are fundamentally restricted by the quality of original data. Moreover, preprocessing procedures involving data screening, feature encoding, and dimensional optimization are complicated, requiring frequent manual intervention and adjustment, which brings high labor and time costs. Owing to the above constraints closely associated with rigorous data processing and model optimization, machine learning inevitably has inherent limitations. Even so, endowed with superior data analysis and intelligent reasoning capacity, it remains the core technical approach for addressing complex computational and predictive problems.

CDs have emerged as a highly promising alternative to traditional luminescent materials due to their unique properties. Studies have shown that the synthesis of CDs depends on various factors, including precursors, reaction methods, solvents, purification methods, pH values, reaction temperatures, and reaction times [15]. Experimental methods used in this process exhibit considerable heterogeneity due to the large number of precursor compounds, different pH levels, and microenvironments, as well as the wide ranges of reaction periods and temperatures. For instance, CDs synthesized under different reaction temperatures and times may exhibit significantly different luminescent properties [159,160]. However, current commonly used CD synthesis methods, such as the hydrothermal method, involve numerous synthesis parameters, resulting in a large and complex search space. Therefore, preparing CDs with specific properties typically requires extensive experiments in the laboratory. In addition, exploring synthesis methods through traditional trial-and-error approaches easily leads to suboptimal results. To improve experimental efficiency and prediction accuracy, ML has been introduced to optimize the experimental process. Currently, ML is used for the controllable synthesis [150,161,162] and quantitative detection of CDs [163,164]. By conducting iterative analysis of experimental parameters, ML can clarify the relationship between the synthesis process and the properties of CDs [165,166].

The construction of ML models is based on the numerical characterization of features. However, in CD synthesis systems, precursors have complex and diverse compositions, and their physicochemical properties are difficult to fully represent through precise numerical encoding. Researchers can systematically review previous literature and select the most frequently used compounds as standard precursors to obtain the most comprehensive dataset. For example, Support Vector Machine Regression (SVR) maps low-dimensional data to a high-dimensional space via kernel functions to fit complex patterns [152]. Key indicators for evaluating the performance of ML models include R^2^ (coefficient of determination), MSE (mean squared error), and MAPE (mean absolute percentage error) [152]. R^2^ is a critical indicator of the degree to which features explain target variability, with values close to 1 indicating a high degree of fit between the model and the data. Meanwhile, MSE quantifies the average squared difference between predicted values and measured values, with lower values indicating more accurate predictions [152]. MAPE is the percentage value of the mean absolute error, reflecting the actual situation of prediction errors; the smaller the MAPE value, the higher the model’s accuracy. To systematically overview the application of machine learning in carbon dot synthesis, Table 4 compares different ML strategies (including XGBoost, DCNN, and ANN-based methods) in terms of input features, prediction outputs, and predictive performance, providing a clear reference for ML model selection in CD synthesis research.

### 4.1. ML-Driven Emission Wavelength Tuning

**Figure 4:**
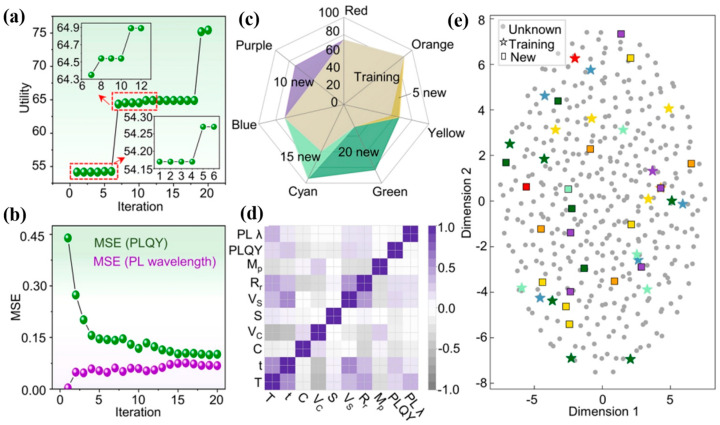
(**a**) Depicts the variation in the unified objective utility of multi-objective optimization (MOO) with design iterations; (**b**) presents the investigation of luminescence colors under novel synthesized experimental conditions, where color ranges are defined by PL wavelength. High PLQY values are achieved for red-, orange-, and blue-emitting materials in the initial dataset, and the MOO strategy enhances the PLQYs of yellow-, purple-, cyan-, and green-emitting materials, respectively, in the subsequent five groups of synthesis experiments; (**c**) shows the mean squared error (MSE) between the predicted and measured values of target properties; (**d**) displays the covariance matrix between eight synthesis parameters and two target properties, namely PLQY and PL wavelength; (**e**) illustrates the two-dimensional t-distributed stochastic neighbor embedding (t-SNE) plot of the entire search space, in which unexplored conditions, training set conditions, and explored conditions are marked by circular dots, star-shaped dots, and square dots, respectively, with the latter two types of data points colored according to the measured PL wavelength [167].

In the preparation of fluorescent CDs, precursor combinations are one of the core variables determining the emission wavelength. The elemental composition, functional group structure, and mixing ratio of different precursors directly affect the electronic energy levels (HOMO-LUMO energy gap) of the synthesized materials, thereby regulating the emission wavelength [169]. The molecular structure of precursors is the fundamental factor determining the optical properties of CDs. The design principle lies in introducing large π-conjugated systems [170] and heteroatoms (N, S, P, etc.) [169] to narrow the optical band gap and promote the emission redshift to the near-infrared region. For example, the strong p-π conjugation effect induced by the non-bonding electrons of graphitic N facilitates the HOMO-LUMO splitting of the carbon core, thereby leading to a significant reduction in the HOMO-LUMO gap [170], which facilitates the generation of red emission. Nevertheless, conventional methods are plagued by low efficiency and high costs due to massive experimental screening of precursor combinations. By contrast, ML can mine the latent nonlinear correlations [171] between precursor combinations and emission wavelengths to achieve precise prediction of targeted precursors, drastically reducing the material development cycle and enhancing regulation efficiency. Guo et al. [167] innovatively proposed a multi-objective optimization strategy, which seamlessly integrates ML with iterative experiments and precise characterization to construct an intelligent closed-loop optimization system for the hydrothermal synthesis of CDs (Figure 4). Due to the high-dimensional search space, limited experimental data, and, particularly, the nonlinear nature of condition–property relationships, constructing a model that can generalize well to unseen data is challenging. Therefore, a model based on XGBoost was adopted, which has been proven to have advantages in processing relevant material datasets. The authors used parameters such as temperature, reaction time, precursor dosage, and solvent volume as synthesis inputs. Eventually, through only 63 ML-guided experiments and 20 iterations, full-color fluorescent carbon dots with tunable emission from blue to red light were successfully obtained, and the PLQY of all colors exceeded 60%, fully demonstrating the excellent efficacy of ML-driven material design. Yan et al. [165] selected the XGBoost model as the optimal ML model, taking four core parameters for CDs preparation (reaction time, sulfuric acid volume, water, and ethanol) as input data. They successfully predicted four key properties of novel multicolor phosphorescent CDs with high precision: (a) fluorescent (FL) emission wavelength, (b) room-temperature phosphorescence (RTP) emission wavelength, (c) FL Stokes shift, and (d) RTP Stokes shift. Specifically, the model achieved an R^2^ of 0.97 for FL emission wavelength and 0.94 for RTP emission wavelength, indicating a strong correlation between predicted and experimental values. In addition, the R^2^ for RTP Stokes shift was 0.95, further confirming the model’s accuracy in capturing key photoluminescent properties. Although the R^2^ for FL Stokes shift was slightly lower at 0.81, it still reflected a high level of prediction accuracy. The mean absolute error (MAE) ranged from 4.23 to 6.14 nm, and the root mean square error (RMSE) ranged from 4.81 to 8.33 nm, indicating small model errors. Overall, these indicators verify that the XGBoost model can reliably predict photoluminescent properties under novel preparation conditions. Red-emitting CDs possess unique imaging characteristics in the biomedical field; however, their conventional synthesis remains a significant challenge. ML can enhance the processing of precursor data, facilitating the accurate prediction of red emission. For example, Luo et al. [161] collected 151 synthesis data points from numerous CD synthesis studies to construct a dataset for the ML model, which can be used to infer new CD synthesis conditions and predict whether the synthesis product is red-emitting CDs. The data processing of the learning model consists of four parts: first, the XGBoost model for feature extraction; second, the one-hot encoding technique for encoding the extracted feature combinations; third, the principal component analysis (PCA) method for dimensionality reduction in the encoded features; and finally, the logistic regression model to infer new CD synthesis conditions and predict whether the product is red-emitting CDs. The results showed that the model had excellent prediction performance, achieving an F1-score of 0.94 and an area under the curve (AUC) of 0.94 in 10-fold cross-validation.

**Figure 5:**
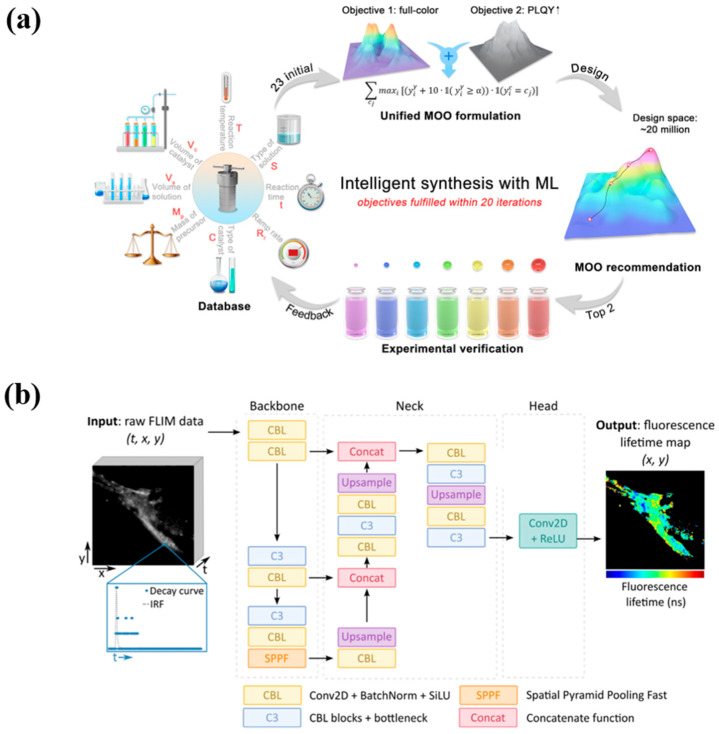
(**a**) Workflow of ML-guided synthesis of CDs with superior optical properties [167]; (**b**) Deep learning for fluorescence lifetime predictions enables high-throughput in vivo imaging [173].

DL is an important branch of ML methods based on ANNs and representation learning, emphasizing the depth of the network, typically consisting of five, six, or even more than ten hidden layers [172] and focusing on mapping samples from the original feature space to a new feature space through hierarchical feature transformation, thereby more effectively completing classification or prediction tasks [152]. For instance, the Multi-Layer Perceptron (MLP) [172], as a fundamental ANN, has laid the basic framework for neural networks through its fully connected hierarchical structure; these algorithms can directly learn patterns from raw data, exhibiting strong advantages in processing large-scale unstructured data and becoming the core pillar of deep learning, which makes them suitable for handling large datasets, providing powerful support for high-throughput screening of CDs and intelligent synthesis control, and promoting the transformation of fluorescent CD research toward data-driven depth. Keras 2.x is an open-source Python-based neural network library designed specifically for rapid experimentation with deep neural networks, featuring user-friendliness, modularity, and high scalability. For example, Wang et al. [155] adopted a one-dimensional (1D) DCNN model based on the Keras framework. Taking synthesis parameters (e.g., precursors, reaction temperature) as inputs, this model was designed to predict the optical properties of CDs, including spectral characteristics and fluorescence color under UV irradiation, and it was trained on experimental data from 170 CD-related studies. Input information is transmitted through multiple hidden layers, and the output layer generates feature vectors that characterize the optical properties of CDs. This model showed good fitting with the training set, with a correlation coefficient of approximately 0.82 for the validation set. Owing to its flexibility in processing multi-component datasets, the ANN was selected as the core machine learning model for predicting the optical properties of CDs. Senanayake et al. [166] employed three machine learning models to predict the emission wavelength of CDs. Specifically, M1 was designed as a base model, which was a regression-optimized artificial neural network ensemble model with the ensemble size k = 4, and it directly realized wavelength prediction by taking spatiotemporal depth features (STD Features) as the input variables. In contrast, M2 and M3 were constructed as two-stage hybrid classification-regression models, whose core innovation lies in the introduction of “luminescent color” as an intermediate feature. The prediction process of M2 and M3 consists of two sequential steps: first, an artificial neural network classification model was utilized to predict the luminescent color of CDs; then, combined with STD Features, a k-value artificial neural network ensemble model was adopted to conduct regression prediction for the emission wavelength. Experimental results demonstrated that the prediction performance of these two-stage hybrid models was significantly superior to that of both the standard artificial neural network and the one-stage artificial neural network regression model. Figure 5 further validates the effectiveness of these data-driven strategies, including the iterative performance improvement, feature correlation patterns, and sample distribution of the ML/DL models, confirming their potential to guide high-throughput screening and intelligent synthesis of CDs.

### 4.2. ML-Assisted Prediction of the Fluorescence QYs

**Figure 6:**
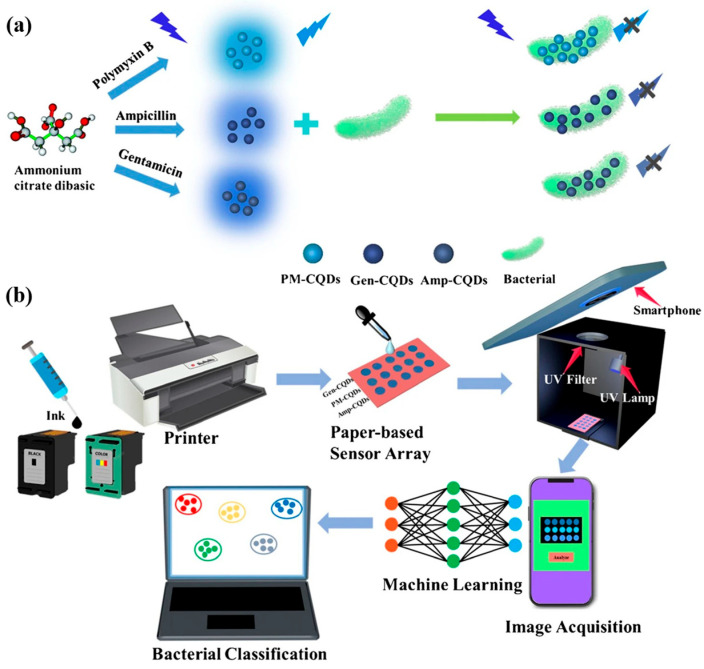
(**a**) Mechanism of bacterial detection based on CDs. (**b**) Schematic of fabrication and bacterial recognition process of the fluorescence sensor-array platform [179].

ML facilitates the quantitative prediction of the PLQY of CDs by mining the intrinsic correlation between CD synthetic parameters and PLQY via model training [168], converting the complex PLQY regulatory mechanism into a data-driven statistical modeling problem. Given that the PLQY of CDs is synergistically modulated by multiple preparative factors with intricate nonlinear coupling effects, conventional techniques fail to achieve quantitative correlation analysis [167]. In contrast, machine learning models, trained on large-scale matched datasets of synthetic parameters and PLQY, can automatically extract key features and capture cryptic microscale regulatory rules through nonlinear fitting, enabling high-precision prediction of PLQY for uncharacterized CDs. This approach circumvents manual feature engineering and markedly reduces the reliance on sophisticated quantum mechanical simulations and labor-intensive repetitive experiments [174]. Han et al. [174] introduced ML into the synthesis of high-quantum-yield carbon quantum dots. The study selected ethylenediamine (EDA), precursor dosage, reaction temperature, heating rate, and reaction time as the input synthesis parameters, and employed the XGBoost-R model to predict and optimize the PLQY of CDs. Eventually, CDs with intense green fluorescence emission were obtained, with a QY as high as 39.3%. These CDs can be applied for Fe^3+^ ion sensing in solutions, exhibiting a wide linear response concentration range and an LOD of 0.039 mol·L^−1^, which demonstrates high sensitivity and selectivity. Chang et al. [16] selected 10 common biochar production wastes as raw materials, with the types of farm wastes; contents of cellulose, hemicellulose, lignin, ash, moisture, N, C, and C/N ratio; pyrolysis temperature; and residence time as preparation parameters (Figure 6). A total of 480 CD samples were extracted from experiments with different combinations of preparation parameters, and the dataset of CD samples was randomly divided into a training dataset and a validation dataset at an 8:2 ratio. The Gradient-Boosting Decision-Tree Regression (GBDT-R) model was used to predict the PLQY of CDs samples. Experimental results showed that the relative error range between the predicted values and the experimental QY values was 0–4.6%, demonstrating that ML can predict the QYs of CDs produced during biochar production from biomass. Ju et al. [175] established a database covering more than 4300 solvated organic fluorescent dyes and 3000 different compounds and designed and trained a stacked model (GBRT/FSD_CDK). Four ML models were used as base learners and a linear regressor as the meta-learner, with total molecular charge, number of conjugated double bonds, and solvent polarity as input parameters. The model aimed to efficiently and accurately predict the emission wavelength and PLQY, complementing quantum mechanical calculations with ML statistical learning methods. Ultimately, the mean absolute error (MAE) of PLQY was significantly 0.13, and the MAE of emission energy was 0.080 eV. Dolenko et al. [176] trained a Multi-Layer Perceptron with a single hidden layer (where the number of neurons ranged from 2 to 128) by using precursor ratios over a wide range, temperature, and reaction time as synthesis parameters, and by training on the QY values of 343 synthetic samples with different synthesis parameters. According to the predicted optimal synthesis parameters, the predicted QY was as high as 99.1%, with a maximum absolute deviation of 3.45% and a standard deviation of 2.14% between the predicted and experimental values. The results indicated that applying MLP to find the optimal parameters for CD synthesis is highly effective when a given QY value is targeted. Nguyen et al. [177] applied ML to guide the synthesis of CDs derived from banana peel agricultural waste via pyrolysis. In this work, precursor type, precursor-to-banana-peel mass ratio, pyrolysis temperature, reaction time, and initial C/N elemental content were selected as input parameters, with the fluorescence intensity of CDs as the prediction target. By integrating central composite design with a Random Forest model, they realized systematic optimization of the synthesis process and high-accuracy prediction of optical properties, delivering a cross-validated R^2^ of 0.97. The resultant CDs exhibited high fluorescence intensity and a quantum yield of approximately 40%. Furthermore, both experimental results and ML analysis confirmed that carbon content and thermal treatment conditions are the primary factors governing the fluorescence performance of biomass-derived CDs [177]. To address the challenges of numerous reaction parameters and high uncertainty in product performance during carbon dot preparation, Xu et al. [178] employed ML to guide the microwave-assisted synthesis of blue-emissive CDs. After optimizing the synthetic parameters and reaction conditions under ML guidance, the QYs of the obtained CDs were approximately 200% higher than the average value of samples prepared without ML assistance. The resultant CDs can serve as a fluorescent probe for monitoring H_2_O_2_ in human teeth, exhibiting a good linear relationship between fluorescence response and H_2_O_2_ concentration in the range of 0–1.1 M with a limit of detection of 0.12 M, enabling effective detection of residual H_2_O_2_ after tooth bleaching. Overall, the prediction error of state-of-the-art ML models for the QYs of CDs can be steadily kept within a narrow range. Ensemble learning models such as GBDT and XGBoost deliver remarkable prediction accuracy, with the root mean square error lower than 0.02 and a low mean absolute percentage error. This level of accuracy facilitates the rapid screening and optimization of synthetic parameters for carbon dots and greatly cuts down experimental trial-and-error costs, which fully demonstrates the practical value of ML in the precise modulation of QYs (Figure 5).

## 5. Application of CDs

### 5.1. Sensing

#### 5.1.1. Conventional Detection

The continuous accumulation and contamination of heavy metals pose severe hazards to the natural environment, impairing the environmental quality of soil, water bodies, and the atmosphere. Timely monitoring of heavy metal contents in the environment is of great significance for formulating corresponding environmental protection measures and reducing risks to ecosystems and human health. The electrostatic adsorption, coordination, and other interactions of heavy metal ions with CDs in solution can affect the fluorescence properties of CDs, thereby achieving the purpose of pollutant detection. At present, CDs exhibit high sensitivity for the detection of heavy metal ions such as Cu^2+^, Hg^2+^, Ag^+^, Cr^3+^, Fe^3+^, and Mn^2+^ [180]. Javeria et al. [114] synthesized fluorescent carbon quantum dots (QY = 56%) from peanut shells through the synergistic effect of urea and boric acid. The prepared CDs showed remarkable sensitivity for on-site visual detection of Ag^+^ and Ba^2+^ in aqueous solutions, with LODs of 0.17 nM and 0.2 nM, respectively. Hafez et al. [181] prepared cerium-doped carbon dots (Ce-CDs) via the pyrolysis of citric acid. Ce-CDs exhibited selective fluorescence-quenching behavior toward Fe^3+^, with good sensitivity and a low LOD of 0.06 μM. They achieved high accuracy in the measurement of Fe^3+^ in tap water samples, with a recovery range of 104.5–107.2%, and could distinguish between the Fe^3+^ and Fe^2+^ oxidation states. Ngara et al. [182] produced highly fluorescent carbon nanodots from pitaya peels, which were used as probes for the detection of Zn^2+^ ions. Notably, with an increase in Zn^2+^ ion concentration, the PL intensity of these CDs was quenched after coordination with Zn^2+^ ions. The quenching of PL intensity of these CDs via Zn^2+^ chelation ultimately resulted in an LOD of 3.2 μM. Kumar et al. [183] prepared highly photoluminescent CDs from mustard pods via calcination. The CDs exhibited high selectivity and sensitivity for Fe^3+^ fluorescent sensing (LOD = 0.042 µM), with recovery rates over 96% in real water samples, providing new strategies for the resource utilization of natural biocompatible raw materials. A. Rezk et al. [184] prepared functionalized, amorphous and uniformly dispersed CDs from sustainable Aloe vera gel through a green one-step hydrothermal method with systematic parameter optimization. The resultant CDs acted as fluorescent probes for wide-range temperature sensing, pH detection, and highly selective Fe^3+^ assay, achieving a low LOD of 16.15 nM.

Food safety has become a major global concern, and the rapid detection of food nutritional components, contaminants, and related risk factors has attracted increasing attention. In the production of high-quality food, food ingredients, condiments, and food additives are widely used to improve the appearance, surface properties, and preservability of food, but the excessive use of condiments poses significant risks to human health and food safety. Biogenic amines [185], as nitrogen-containing organic basic compounds released during food spoilage, have become core characteristic indicators for monitoring food freshness. Fluorescent detection methods boast the technical advantages of high sensitivity, simplicity, and rapidity; in particular, methods based on the mechanisms of fluorescence quenching or probe recovery exhibit broad application prospects in the field of food detection. Gu et al. [186] used citric acid as the carbon source and halogenated homologues of o-phenylenediamine as ligand precursors. CDs doped with different halogens exhibit significant color changes from blue to red due to differences in the key fluorophore molecules (pyridone derivatives). Based on the pH-responsive characteristics of color changes, a putrescine-sensing probe was developed. Meanwhile, visual and quantitative food freshness monitoring can be performed via smartphones. Zhang et al. [187] synthesized highly efficient red-emissive carbon dots (R-CDs) using citric acid, polyethyleneimine, and benzyl via a simple solvothermal method. The fluorescence of R-CDs can be effectively quenched by sunset yellow. This probe has a linear range of 0.085–11.31 μg·mL^−1^, an LOD of 0.026 μg·mL^−1^, a recovery rate of 91.2–122%, and an RSD of 1.0–3.5%. Therefore, R-CDs can be used as fluorescent probes for the detection of Sunset Yellow in food. Mansour et al. [188] synthesized NS-doped carbon dots (NS-CDs) using citric acid and D,L-methionine as precursors, which emit bright blue fluorescence at an emission wavelength of 440 nm with a PLQY of 38.68%. In the range of 25–250 μg·mL^−1^, the fluorescence intensity shows a linear relationship with the increase in monosodium glutamate (MSG) concentration, with an LOD of 6.20 μg·mL^−1^ and an LOQ of 18.79 μg·mL^−1^. Most current fluorescent sensors based on carbon quantum dots rely only on changes in the intensity of a single emission peak. This method is prone to errors due to interference from background noise, matrix effects, instrument parameters, and the concentration distribution of fluorescent probes. These defects are addressed by using ratiometric fluorescent sensors, which can achieve a lower LOD and a wider linear range. Black pepper is one of the most commonly used spices, containing alkaloids and porphyrins. Tang et al. [189] selected it as a raw material and effectively prepared ratiometric CDs with two emissions (520 and 668 nm) under single-wavelength excitation at 415 nm via a solvothermal method. The formation of CDs-Fe^3+^ complexes led to the main quenching of near-infrared (NIR) emission at 668 nm, and the redox reaction between Fe^3+^ and ascorbic acid (AA) restores the fluorescence. Therefore, the obtained CDs were developed as an “off–on” mode NIR ratiometric nanoprobe for ascorbic acid determination, with excellent selectivity and high sensitivity. The LOD and LOQ are 0.30 μM and 0.99 μM, respectively, with an RSD below 4.6% and a recovery rate ranging from 94.6% to 106.5%.

The concentration, distribution, and dynamic fluctuations of ions in living cells are critically involved in regulating core physiological processes including cell proliferation, signal transduction, and energy metabolism. Dyshomeostasis of intracellular ions is closely implicated in the pathogenesis and progression of various diseases. Highly sensitive and selective detection of intracellular ions serves as an important foundation for elucidating the molecular mechanisms of life activities and facilitating early disease diagnosis. Owing to their superior biocompatibility, excellent photostability, and facile surface functionalization, CDs have emerged as ideal fluorescent probes for intracellular ion sensing. Zhang et al. [190] addressed the challenge of accurate Mn^2+^ quantification in living cells, which limits mechanistic studies of the Mn-mediated cGAS-STING pathway. They developed a novel Mn^2+^-specific fluorescent probe, Mn^2+^-CDs-MIPs, by integrating molecular imprinting with CDs. The probe displays dual emissions at 365 nm (response) and 520 nm (reference) under 290 nm excitation. Mn^2+^ selectively enhances the fluorescence at 365 nm with negligible effect on 520 nm, enabling a ratiometric Mn^2+^ sensor. It exhibits a linear range of 0.8–80 μM and a detection limit of 0.3 μM, with high specificity and salt tolerance, allowing quantitative Mn^2+^ detection in complex biological systems and living cells. Nasrin et al. [191] prepared green-emitting CDs from Strophanthus gratus leaves via a simple hydrothermal method. These CDs show low cytotoxicity to human hepatic WRL68 cells and can serve as fluorescent probes for highly sensitive Cr^6+^ detection (LOD = 34 nM) through fluorescence quenching. Notably, they enable in situ fluorescence imaging of Cr^6+^ in living WRL68 cells and are also applicable to quantitative Cr^6+^ analysis in real water samples. Hu et al. [192] developed green emissive CDs modified with cholesteryl (Chol-GCDs, QYs = 65%) as multivalent stable fluorescent probes for extracellular vesicles (EVs), which are key mediators of intercellular communication. This work addresses the need for accurate EV detection and overcomes limitations of conventional probes, including false positives from hydrophobic dye aggregation and membrane damage by inorganic phosphors. Their 96-well plate-based sandwich-type fluorescent platform enables quantitative EV detection from 10^3^ to 10^6^ particles per mL, with good analytical performance in cell culture supernatants and serum. This platform provides a promising tool for quantitative analysis of cell-secreted EVs and functional studies of intercellular communication and disease-associated EVs. Xu et al. [193] synthesized blue emissive CDs using raspberry as the carbon source. These dots exhibited high selectivity for Cu^2+^ and underwent dynamic fluorescence quenching via electrostatic interaction, successfully achieving intracellular Cu^2+^ detection and providing a reference for the design of carbon-based fluorescent sensors. Wang et al. [194] developed a dual-color CD probe via one-step hydrothermal synthesis for highly sensitive and reversible detection of a superoxide anion radical (O_2_^−^). It has been applied to real-time visual monitoring of O_2_^−^ in normal hepatocytes and hepatic ischemia–reperfusion injury cell models, providing an efficient tool for dynamic analysis of reactive oxygen species in living cells and related pathological studies. Srivastava et al. [195] synthesized multicolor CDs (MCQDs) from mahogany leaves via a one-step hydrothermal method, which detected Fe^3+^ and As^3+^ in water through fluorescence quenching and enhanced dual responses and enabled effective detection of both heavy metal ions in NIH 3T3 cells. Jeeva et al. [196] prepared CDs from curry leaf seeds with strong fluorescence, enabling highly sensitive nanomolar detection of Fe^3+^. The dots show good biocompatibility and are suitable for fluorescence bioimaging in HCT 116 human colon cancer cells.

#### 5.1.2. AI-Assisted Detection

Conventional quantitative detection methods for fluorescent CDs mainly rely on calibration curves between fluorescence intensity and analyte concentration, and this method has obvious limitations. Fluorescence intensity is highly susceptible to the influence of microenvironmental factors, resulting in significant discrepancies in detection results for analytes at the same concentration under different conditions. In addition, analytes, CDs themselves, or interfering substances may all cause overlapping emission spectra, making it difficult to accurately detect target signals and thus posing great challenges to the quantitative detection of fluorescent CDs. However, ML technology enables algorithms to learn and extract feature patterns that are highly correlated with analyte concentration from complex, multi-dimensional fluorescence data, thereby establishing more stable and accurate quantitative prediction mode. This technology effectively overcomes the shortcomings of traditional methods and greatly improves detection accuracy [197,198]. Pang et al. [199] synthesized CDs with high selectivity for Zn^2+^ using microcrystalline cellulose extracted from biological waste as the raw material, and further developed an ML-driven detection framework that combines spectral feature cluster analysis with a lightweight MobileViT architecture to identify and quantify different fluorescent signals. Through the automatic analysis of 650 fluorescence spectra, this intelligent system improved the Zn^2+^ identification accuracy to 82.4% while enabling real-time quantitative detection and analysis. ML optimization allowed the CDs to achieve excellent performance even in complex sweat matrices with multiple interferences (LOD = 0.17 μM). Li et al. [200] designed a sensor array composed of three unique herb-derived nitrogen-doped carbon dots (NCDs). Combined with an ML model, this sensor array exhibited strong anti-interference capability for warfarin identification in unknown samples. In the detection of diluted serum samples, the recovery rate ranged from 90.6% to 100.1%, with an RSD of less than 9.4%. Zhang et al. [201] prepared CDs with high PLQY using a green carbon source (citric acid) via a solvothermal method, and developed a fluorescent array based on four molecularly imprinted polymer (MIP) sensing units. Differentiated recognition sites were constructed by combining high-quantum-yield CDs with multi-template imprinting technology, which generated specific fluorescence-quenching patterns through charge transfer mechanisms. Using a linear discriminant analysis (LDA) ML algorithm, this array achieved high-precision identification (98.96% accuracy) and sensitive quantification (LOD: 0.026 μg·mL^−1^) of 12 structurally similar sulfonamide antibiotics (SAs). This method provides an effective strategy for the screening of trace SAs and can be extended to the multi-component identification of other structurally similar pollutants. Zhai et al. [202] prepared multicolor fluorescent CDs using citric acid and other precursors. Based on the color changes generated by the interaction between multicolor CDs and target pesticide molecules, color signal characteristic values were extracted, and visual data acquisition was integrated with an ML model. A dual-source data acquisition strategy enabled efficient qualitative identification and quantitative detection of multiple pesticides, with a pesticide classification accuracy of up to 99.3% and robust quantitative prediction accuracy for pesticide concentration detection (R^2^ ≥ 0.8946). ML has injected powerful intelligence into the quantitative detection of fluorescent CDs. Rawat et al. [203] constructed an inkjet-printed paper-based fluorescent sensor array using CDs modified with three different antibiotics. Based on a bacteria-induced aggregation-caused fluorescence-quenching effect, combined with smartphone imaging and machine learning algorithms, this platform can accurately distinguish five bacterial strains over a wide detection range of 10^3^–10^7^ CFU/mL. Owing to its low cost, facile fabrication, and excellent portability, the sensor presents great application potential for on-site and rapid bacterial detection. To address the challenge of accurate discrimination of dihydroxybenzene isomers caused by structural similarity and cross-sensitivity, Li et al. [204] constructed a fluorescent sensor array based on five citric acid-derived CDs engineered via distinct nitrogen doping pathways. Coupled with five supervised ML models—linear discriminant analysis (LDA), k-nearest neighbor (KNN), logistic regression (LR), naive Bayes (NB), and artificial neural network (ANN)—the array achieved classification of pure isomers and their binary and ternary mixtures. The artificial neural network delivered the best performance, yielding an overall recognition accuracy of 97.28% at a concentration as low as 1 μM, and the reliability of the method was confirmed by real-sample validation and blind tests. To meet the urgent demand for rapid detection of toxic metal ion contamination in aqueous environments, Jesús Mejía-A et al. [205] fabricated a dual-mode nanosensor based on citric acid-derived nitrogen-doped carbon dots (N-CDs) for simultaneous detection of multiple metal ions via fluorescence and colorimetric readouts. The fluorescence mode delivers a nanomolar-level detection limit, while the colorimetric response, clearly distinguishable under natural light, supports both rapid visual inspection and smartphone-assisted digital colorimetric analysis. Integrated with ML algorithms, the system realizes automatic identification of metal ion species and quantitative concentration determination, with a classification accuracy of over 97%. This intelligent detection platform operates without complex instrumentation and boasts the merits of portability, high sensitivity, and eco-sustainability, demonstrating prominent application potential for on-site heavy metal detection. By mining hidden deep correlations in multi-dimensional fluorescence data, it provides a robust tool for achieving highly accurate, reliable quantitative analysis of CDs applicable to complex real-world scenarios. Table 5 further validates the practicality of ML-assisted CD-based detection strategies, showing high accuracy in diverse analytical tasks ranging from ion recognition to pharmaceutical analysis, thus highlighting their optimization of conventional detection systems.

The most common issue with conventional sensors remains time consumption, coupled with the lack of user-friendly on-site monitoring capabilities. With the popularization of smartphones, their digital imaging functionality has made them excellent analytical platforms for developing point-of-care (POC) detection sensors. To date, numerous biometric detection platform methods using smartphones have been reported (Figure 6). For example, Thonghlueng et al. [17] selected citric acid as the precursor to synthesize blue fluorescent CDs via a microwave method and further developed a dual-response fluorescent sensor for the detection of cytosine and 5-methylcytosine in urine. The sensor exhibited LOD of 43.4 μM and 74.4 μM for cytosine and 5-methylcytosine in urine, respectively, with satisfactory recovery rates (103.5–115.8%). In addition, a smartphone integrated with ML functionality was developed, which can be effectively applied to the determination of methylation levels (0–100%). These results demonstrate a simple and effective method for detecting methylation levels in urine, which is of great significance for future clinical prediction. Feng et al. [217] prepared dual-chromatic CDs using fresh aloe vera leaves and neutral red, and constructed a portable handheld sensor based on Hg^2+^-mediated ratiometric fluorescent CDs for the ultra-rapid (30 s) intelligent detection of glutathione (GSH). This sensor enables on-site monitoring of GSH with an LOD of 1.84 μM, providing a valuable and efficient tool for rapid on-site chemical analysis and intelligent point-of-care diagnosis. The combination of smartphones with ML-assisted fluorescent CDs represents a cutting-edge direction for advancing portable, intelligent, and low-cost detection technologies. This integration fully leverages the popularity, sensing [218], and computing capabilities of smartphones, as well as the high sensitivity, tunable fluorescence properties, and biocompatibility of CDs; further, ML is employed to process complex data and improve analytical accuracy. Chang et al. [16] synthesized blue/yellow emissive ratiometric fluorescence using ink via a hydrothermal method, and designed a portable AI-driven handheld sensor. The intelligent platform consists of a high-throughput paper sensor loaded with CDs, a customized 3D-printed accessory, and a smartphone with ML functionality. In real-time mode, an open-source ML algorithm is deployed in a self-programmed applet on the smartphone to directly output the amoxicillin concentration of samples; subsequently, a fitting equation is selected via online analysis to achieve intelligent on-site analysis. This platform enables rapid detection of amoxicillin over a wide range of 5 μM to 1000 μM, with an acceptable LOD of 2.8 μM. Finally, the test results of samples can be directly read via the AI-driven handheld sensor. Liu et al. [219] prepared blue fluorescent CDs using fresh orange leaves and designed a three-color ratiometric fluorescence optical device integrated with an ML-assisted smartphone to visually monitor tetracycline antibiotics (TCs), which can meet the demand for on-site real-time detection. The determination of four TCs in milk via the standard addition method and the verification of the detection method using a portable device and smartphone platform showed consistent results, confirming that this technology can accurately quantify TCs in milk and provide reliable support for food safety monitoring. More importantly, compared with fluorescent probes reported in the previous literature, the visual detection of TCs provides rich and extensive color changes, offering new possibilities for on-site real-time rapid detection of antibiotics. Lu et al. [211] also developed an optical monitoring device combining a smartphone with a multicolor ratiometric fluorescence colorimetric paper sensor for the detection of Hg^2+^ and S^2−^ in water and seafood, with LODs of 0.002 μM and 1.488 μM, respectively. Meanwhile, it integrates a hydrogel intelligent film to monitor food freshness via headspace sampling. The smartphone app, programmed with a multi-model deep learning algorithm, is dedicated to selecting the most matching fitting method based on the characteristics of each dataset, establishing a more accurate linear relationship between chromatic signals and the concentration of detected substances, and enabling the detection of the maximum number of samples. Collectively, the aforementioned studies confirm the feasibility and superiority of integrating smartphones with CD-based sensing systems, while Table 6, by systematically compiling the construction materials, target analytes, and key performance parameters (including linear ranges and limits of detection, LODs) of representative works, further quantitatively validates the versatility and reliability of such portable detection platforms for high-sensitivity, wide-range analysis in complex sample matrices.

### 5.2. Biomedical Field

**Figure 7:**
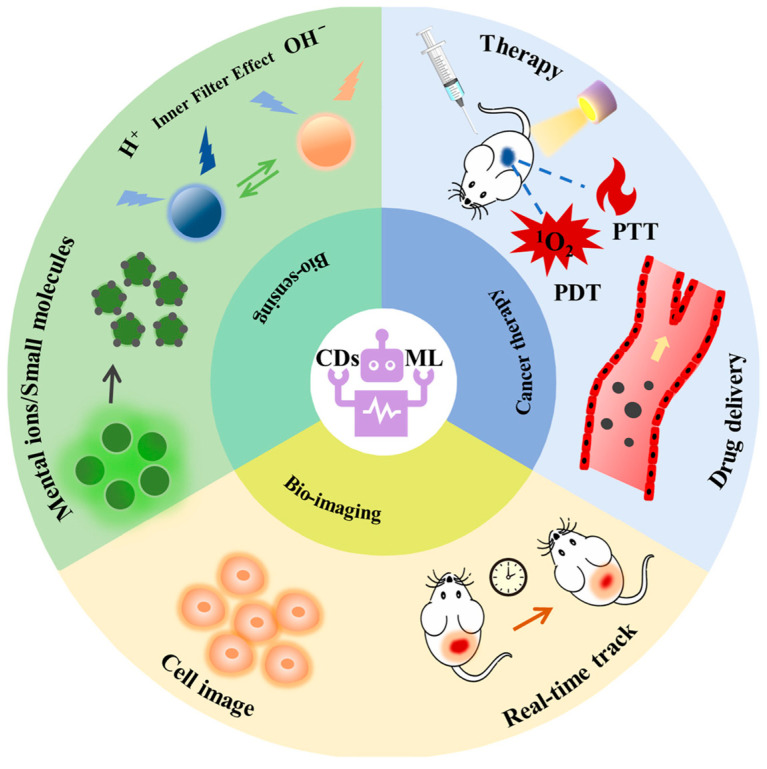
A schematic illustration of biological applications for CDs combined with ML [246].

CDs possess unique optical properties, a large surface area, multicolor emission profiles, small size, low cytotoxicity, excellent biocompatibility, and superior photostability, which endows them with extensive applications in the biomedical field. CDs produced via various technologies have been employed in a wide range of applications, including bioimaging [238,239,240], biosensing [241], chemical or fluorescent sensing [68], nanomedicine [242,243,244], and drug delivery [245] (Figure 7).

#### 5.2.1. Cell Imaging

**Figure 8:**
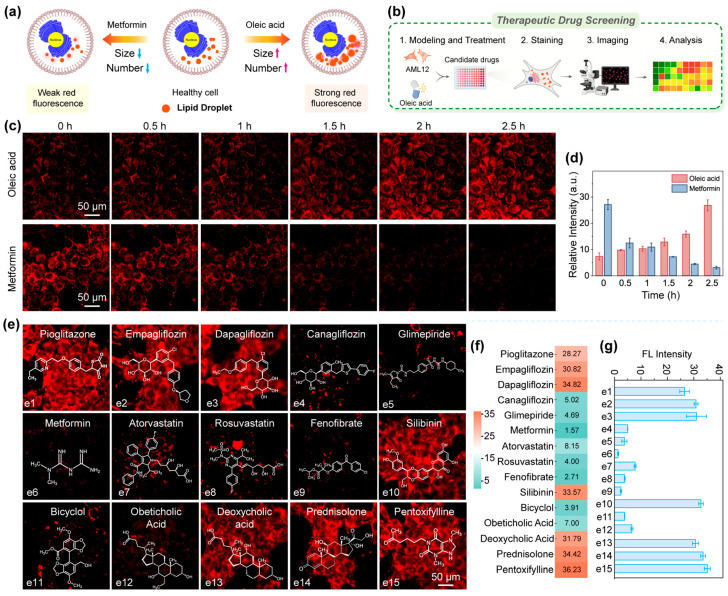
LD imaging and MASLD drug screening by NIR-FCDs. (**a**) Diagram of metformin and oleic acid inhibiting and increasing LDs. (**b**) Schematic diagram of NIR-FCDs for MASLD drug screening. (**c**) CLSM images of AML-12 cells that were incubated with 800 μM metformin or oleic acid for different durations (0 to 2.5 h) and then stained with 50 μg mL–1 NIR-FCDs for 30 min (λex = 514 nm, λem = 550–655 nm). (**d**) Quantitative analysis of the relative fluorescence intensity is displayed in (**c**). (**e**) Confocal images of oleic acid-pretreated AML-12 cells, which were then treated with various drugs, followed by incubation with NIR-FCDs. (**f**,**g**) Quantitative analysis of the relative fluorescence intensity displayed in (**e**). Scale bar: 50 μm [251].

Properties such as low toxicity, biocompatibility, and fluorescence characteristics render CDs more advantageous for the visualization of biological systems in vitro and in vivo [247]. Unlike conventional semiconductor quantum dots, CDs are non-toxic, heavy metal-free, and highly resistant to photobleaching, while also being easily synthesized through green, scalable methods. Progress in size regulation, surface modification, and heteroatom doping has enabled CDs to emit near-infrared light, a key requirement for deep-tissue imaging that reduces autofluorescence to a minimum [248]. For example, Bian et al. [249] synthesized LF-CDs via a one-pot hydrothermal method using citric acid as the precursor, which can detect exogenous and endogenous hypochlorous acid in living cells and enable imaging of intracellular hypochlorous acid during ferroptosis. Fluorescence imaging is a key tool for visualizing the morphology and dynamics of living cells (Figure 8). Govindhan et al. [101] green-synthesized blue fluorescent carbon dots (PS-CDs) from papaya seed biomass waste via a microwave-assisted method. Biocompatibility and imaging capabilities of PS-CDs were confirmed through bioimaging experiments using Caenorhabditis elegans, with fluorescence observed in the pharynx and midbody regions of the nematodes. These results demonstrate that biomass waste can be converted into valuable nanomaterials. Le et al. [9] green-synthesized fluorescent CDs from banana juice via a microwave method, and further synthesized biofluorescent CDs decorated with biodegradable periodic mesoporous organosilica nanoparticles (BPMO), yielding BPMO@CDs. The uptake rate of BPMO@CDs reached 46.74% in MCF7 cells and 17.07% in L929 cells, with stable fluorescence maintained intracellularly. The optical properties of CDs were fully preserved in the composite material, enabling tracer monitoring of the cellular uptake process and highlighting their potential for synergistic applications in bioimaging and chemotherapy. Kirubanithy et al. [250] fabricated N-doped carbon dots (N-CDs) from bamboo stems via a hydrothermal method. The as-prepared N-CDs overcame the limitation of insufficient fluorescence intensity of biomass-derived carbon dots, and simultaneously exhibited a high QY (50.89%), an ultrasmall size of 2.4 nm, as well as excellent hydrophilicity. With enhanced optical properties, reduced cytotoxicity, and integrated merits of various phytochemicals from bamboo stems, the N-CDs enabled bioimaging of various cell types. Furthermore, after conjugation with folic acid (FA), the FA-modified N-CDs realized folate receptor (FR)-mediated targeted imaging of FR-positive HepG2 cancer cells. Owing to their high fluorescence intensity, the N-CDs yielded high-quality imaging under a fluorescence microscope, serving as an excellent alternative to carcinogenic dyes.

Based on their excellent optical properties, CDs can be used to develop robust and accurate cell imaging and real-time tracking technologies, facilitating disease diagnosis and scientific research. The current demand for high-quality CDs is increasingly urgent, and ML can effectively guide the synthesis process of CDs. By applying ML algorithms to CD preparation, researchers have successfully improved the performance of CDs and significantly enhanced the precision and resolution of cell imaging. These breakthroughs mark major advances in the fields of nanotechnology and bioimaging, and are expected to drive the development of more efficient material systems and more reliable diagnostic tools. Luo et al. [161] adopted one-hot transformation for data features and extracted data features using principal component analysis combined with XGBoost. The logistic regression model exhibited good predictive performance for determining whether the synthesized CDs were red-emissive. This model was used to optimize the production process and ensure the most efficient production of high-quality red CDs. Live cell imaging with these CDs demonstrated their low toxicity and applicability for cell imaging.

#### 5.2.2. Drug Delivery

**Figure 9:**
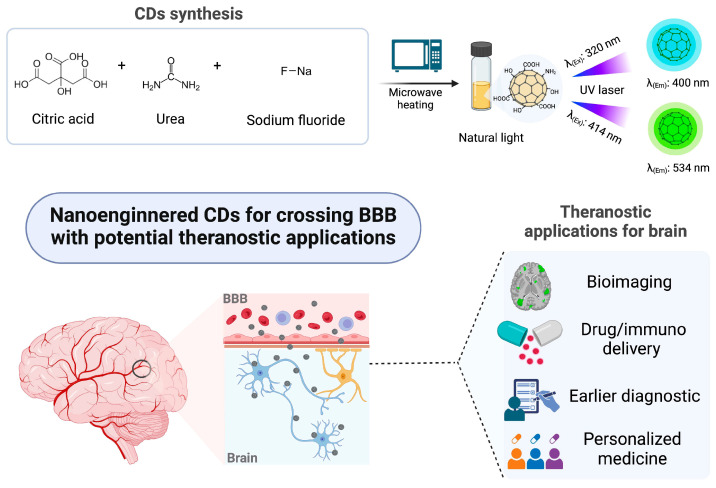
Representation of CDs nanoengineered for crossing the blood–brain barrier (BBB) and their potential as a theranostic nanosystem in neurological diseases [256].

Fluorescent ultrasmall nanoparticles (<10 nm), such as CDs, possess optimal size and low biotoxicity, enabling them to diffuse in complex microenvironments for drug delivery, imaging, and monitoring [252,253]. Multifunctional nanomaterials capable of monitoring the expression of specific biomarkers and serving as carriers for controlled drug delivery are highly desirable [254]. In addition, CDs are rapidly excreted from the human body and are considered environmentally harmless [255]. For example, Araújo et al. [256] synthesized NF-CDs via a microwave-assisted method using citric acid (a green carbon source) as the precursor. NF-CDs successfully crossed the blood–brain barrier (BBB) in a time-dependent manner. Compared with the control transwell system (cell-free), NF-CDs achieved 70% permeability in the fully developed BBB, versus 81% in the control, within only 2 h (Figure 9). This demonstrates that NF-CDs are a very promising nanosystem for brain-targeted bioimaging, nanocarrier-based drug and immunotherapy delivery, early diagnosis, and personalized medicine. Cancer is a multifactorial disease, and combination strategies are most likely to address metastatic tumors and effectively avoid recurrence. Cillari et al. [257] conjugated N, S-doped CDs with indoximod (IND) to develop an innovative nanosystem (CDs-IND). CDs-IND not only retains the advantages of bare CDs, including photoluminescence for self-tracking, but also significantly controls breast cancer progression after intratumoral and intravenous administration of CDs-IND in vivo. The synthesized CDs not only have inherent properties of low toxicity and high biocompatibility but also retain the characteristics of their precursors. In addition to achieving active targeting through receptor–ligand interactions, CDs can realize synergistic photothermal/photodynamic therapy—they absorb light energy and convert it into thermal energy, inducing cancer cell apoptosis and disrupting cancer cell membrane structure. Drug delivery, photodynamic therapy, and photothermal therapy are some of the prevailing applications that exploit the unique properties of CDs, surpassing the established methods, which are responsible for a high degree of fatalities [258]. For example, Zhao et al. [259] synthesized red-emissive carbon dots (5-FICD) using the anticancer drug 5-fluorouracil (5-FU) and the photosensitizer indocyanine green (ICG) via a simple method. The retention property of ICG enables 5-FICD to selectively accumulate in hepatocellular carcinoma cells. Under light irradiation, 5-FICD emits bright red fluorescence, effectively illuminating the intracellular microenvironment, while inducing photothermal and photodynamic effects. The apoptosis rate of hepatocellular carcinoma cells treated with 5-FICD reached as high as 85.93%. Banerjee et al. [260] significantly enhanced the photophysical properties of CDs through the covalent conjugation of pepsin with CDs, leveraging the intrinsic oxidative stress-inducing ability of pepsin. ML modeling was performed using Orange software 32 to optimize the synthesis conditions. The input data included eight feature variables and one target variable of yield grade. The feature variables included categorical variables (salt type and desolvating agent type) and numerical variables (pepsin percentage, salt concentration, pH value, desolvating agent volume, desolvating agent dipole moment, and desolvating agent dielectric constant). A seamless integration of bioimaging and reactive oxygen species (ROS)-mediated photodynamic effect was achieved using a simple LED light source. This technology exhibits significant targeting effects on cancer cells and microbial pathogens—taking THP-1 cells (derived from human monocytic leukemia and widely used in nanoparticle uptake studies) as an example, their performance in bioimaging and drug delivery studies verified the effectiveness of this technology. In addition, CD-pepsin nanoparticles exhibit excellent structural stability and sustained drug release capacity, further highlighting their potential in multifunctional theranostic applications.

### 5.3. Other Applications

#### 5.3.1. Light-Emitting Devices

**Figure 10:**
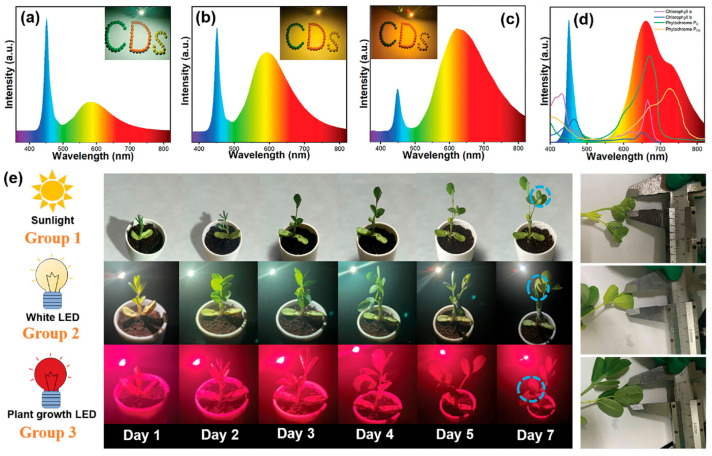
(**a**–**c**) PL emission spectra of the white LEDs based on Y-CDs, Y-/R-CDs, and Y-/R-/DR-CDs, respectively (inset, the object color under prepared white LEDs). (**d**) PL spectrum of the plant growth LED device based on DR-/NIR-CDs and absorption spectra of chlorophyll A and B and phytochrome P_R_ and P_FR_. (**e**) Panoramic images of peanut growth for 7 days under the conditions of sunlight, white LED, and plant growth LED [267].

Light-emitting diodes (LEDs) [261], as a new generation of green light sources, have been widely applied in various fields of daily life due to their advantages such as energy saving, environmental friendliness, long lifespan, and cold light emission. Currently, the color conversion materials of LEDs mainly include phosphorescent materials, semiconductor quantum dots, and fluorescent materials (Figure 10). Among them, phosphorescent materials tend to aggregate at the micrometer scale, limiting the color conversion efficiency of LEDs; semiconductor quantum dots contain heavy metals such as Cd and Cr, which pose serious hazards to the environment and human health [262]; traditional phosphors require a calcination temperature of approximately 1000 °C and rely on rare earth elements. In contrast, CDs are excellent candidates, exhibiting excellent environmental friendliness and biocompatibility due to their simple synthesis process and green preparation conditions. Yong et al. [263] prepared single-component and dual-component solid-state white-light CDs from biological pollutant cyanobacteria via a microwave-assisted hydrothermal method, with the solid-state PLQY of single-component CDs reaching as high as 41.8%. White-light and red-light CDs were used as phosphors to fabricate fluorescent films and cold, pure, and warm white-light and red-light LEDs, which exhibited good photobleaching resistance and temperature stability, with color rendering indices (CRIs) of 84.4, 85.7, and 61.5, respectively. Niu et al. [264] prepared CDs with Alternaria sp. as the carbon precursor via a one-step hydrothermal method, with the emission spectrum extended to cover the entire visible light region (400–700 nm) and the PLQY enhanced to 22%. Light-emitting devices based on W-CDs realized standard white-light emission. Additionally, this study proposed an eco-economic “waste–control–waste” strategy for invasive plant management and pioneered a green synthesis route for biomass-derived white CDs and their optoelectronic integration. Mai et al. [265] synthesized red-emissive CDs (R-CDs) with chlorophyll molecules embedded in a carbon-based matrix using ethanol extracts of bougainvillea leaves as the green carbon source via a solvothermal method. Devices based on R-CD phosphors can partially convert the 410 nm light emitted by LED chips to the red–far-red light region, thus providing a more cost-effective single-chip LED solution for agricultural lighting. Developing new alternative methods to recycle PET waste, which has important implications for reducing landfilling and CO_2_ emissions, has long been a research hotspot in industry and academia. Ma et al. [266]. prepared nitrogen-doped carbon dots (NCDs) using PET waste as the precursor. The maximum excitation and emission peaks of the obtained NCDs are situated at 360 nm and 470 nm, respectively, with a PLQY of 48.16%. The NCDs were dispersed in polymethyl methacrylate (PMMA) and subsequently loaded onto 365 nm and 430 nm LED chips separately. The as-fabricated LED devices emitted yellow light (CIE coordinates: (0.55, 0.44), correlated color temperature: 2018 K) and warm white light (CIE coordinates: (0.37, 0.31), correlated color temperature: 3783 K), respectively.

#### 5.3.2. Catalysis

**Figure 11:**
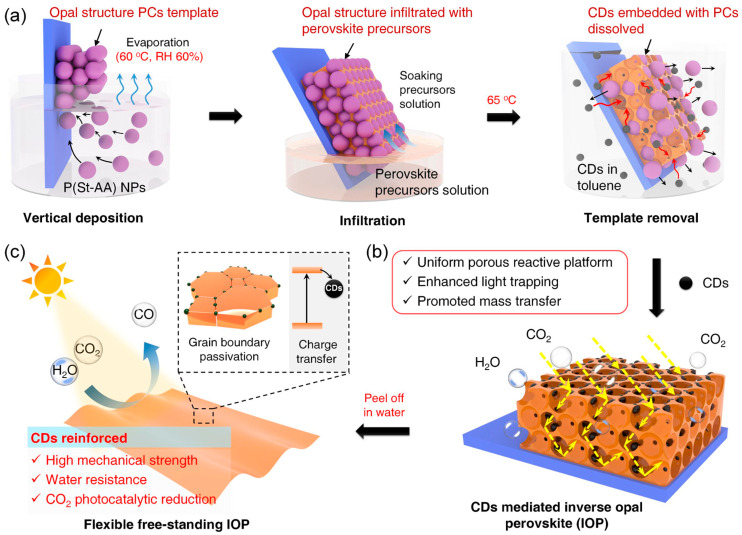
Schematic of synthesis process of flexible CDs/CsPbBr_3_ IOP film for enhanced photocatalytic CO_2_ reduction. (**a**) Fabrication process of CD-embedded IOP via vertical deposition of opal PC template, infiltration of perovskome precursors, and template removal. (**b**) Structural merits of CD-mediated IOP, including a uniform porous reactive platform, enhanced light trapping and promoted mass transfer, as well as the preparation of flexible free-standing IOP membrane via water peeling. (**c**) Photocatalytic CO_2_ reduction mechanism and performance advantages of CDs-reinforced IOP, featuring grain boundary passivation, charge transfer promotion, high mechanical strength, water resistance, and efficient CO_2_ photoreduction [275].

In recent times, several researchers have been focusing on CDs with enhanced surface area-to-volume ratio, biocompatibility, morphology, band gap, and efficient adsorption properties for the removal of toxic dyes [268]. As promising photocatalysts in the domain of photocatalysis (Figure 11), CDs exhibit distinctive advantages, including efficient utilization of visible light, fast charge carrier migration, and adjustable energy level structures [269]. Photocatalysis [270] refers to the material transformation process induced by the synergistic effect between photocatalysts and light. The key step of this process is the light-induced separation of charge carriers. An ideal photocatalyst can not only provide a broad absorption spectrum, but also achieve highly efficient separation of photogenerated charge carriers. Such charge separation relies on carbon dots absorbing light with sufficient energy to surmount the band gap of the nanoparticles [271]. Heteroatom doping and surface functionalization of carbon dots can reduce their energy band gap [269]. Therefore, modifying conventional photocatalysts with CDs can enhance the light absorption capacity and charge separation efficiency of the materials, thereby significantly boosting their overall photocatalytic activity. A number of recent studies have validated that the photocatalytic performance of various CD-modified composite photocatalysts has been remarkably improved. For example, Vijeata et al. [268] green-synthesized highly fluorescent CDs using Azadirachta Indica leaves, which exhibit excellent biocompatibility and a high PLQY of 42.3%. Light-triggered photocatalytic experiments showed that after 70 min of mercury lamp irradiation, the degradation rates of the developed CDs for methylene blue (MB), malachite green (MG), rhodamine 6G, and methyl orange (MO) reached 90.73%, 98.25%, 52%, and 6.13%, respectively. Wechakorn et al. [272] developed multifunctional CDs from cassava peels via a green hydrothermal method; these CDs exhibited efficient photocatalytic degradation of methylene blue (72%) under various light sources and also showed strong antioxidant activity with a scavenging inhibition rate of 82% at a concentration of 10.0 mg·mL^−1^. To ensure biological and environmental safety, there is an urgent need for rapid and efficient treatment technologies for dye-contaminated wastewater. Photocatalysts based on natural precursors, especially CDs, are currently widely used for the remediation of dye-contaminated wastewater. Singh et al. [273] prepared nitrogen-doped carbon dots (N-C-CDs) via a simple pyrolysis method using garlic as the precursor, which were applied for the photocatalytic purification of crystal violet (CV) wastewater. In photocatalytic performance tests, 0.06 g of the catalyst achieved a degradation rate of 99.13% for 40 ppm CV dye under UV irradiation for 80 min at pH = 5. Notably, this material also exhibited excellent reusability with an efficiency >95% after 5 cycles. Developing novel waste-recycling strategies has become a feasible solution to overcome environmental pollution. Wang et al. [274] synthesized blue fluorescent CDs using turbine blades as the carbon source via hydrothermal treatment, achieving a relatively high PLQY of 29.9% and a utilization rate of 40%. Notably, these CDs can also serve as photocatalysts for methylene blue degradation, with a degradation efficiency of 64% after 40 min of irradiation. Comprehensive studies have confirmed that CDs have been successfully applied as photocatalysts in numerous fields, and they sometimes even outperform the more widely used traditional methods. The continuous development of CD technology is expected to drive an environmentally friendly and low-resource-consumption model for basic chemical transformations by coupling solar energy (a renewable energy source), providing sustainable solutions for daily chemical reaction processes [275]. Archana et al. [276] developed a CD–morin (CD–MO) dual-emission fluorescence sensor using CDs derived from dragon fruit peel waste. The sensor permits highly sensitive Cr^6+^ detection in real samples and photocatalytically reduces toxic Cr^6+^ to non-toxic Cr^3+^ through photoinduced electron transfer, realizing the integration of detection and remediation functions. Electrochemical detection and photocatalytic degradation of Cr^6+^ within a single material platform are demonstrated.

Electrochemical [277] reactions are of great significance for electrochemical energy conversion devices that directly convert electrical energy into chemical energy or vice versa. However, the limited electrocatalytic activity and poor stability of catalysts have seriously hindered the development of renewable energy-related conversion devices [278]. CDs possess the characteristics of non-toxicity, abundance, and low cost, along with unique electron transfer capability and a large specific surface area, which endow them with excellent performance in electrochemical reactions [279]. More importantly, heteroatoms in CDs can effectively regulate charge distribution and promote electron transfer through internal interactions, which is crucial for improving electrocatalytic performance [280]. Obtaining electrocatalysts from biomass residues is a feasible strategy that can minimize the environmental impact of electrode production and reduce manufacturing costs. Alemany-Molina et al. [281] successfully prepared Fe-N-C catalysts by mixing almond shell residues as the precursor with dicyandiamide and an appropriate amount of FeC_2_O_4_, which were used as cathode electrocatalysts for alkaline direct methanol fuel cells (DMFCs). Wanchan et al. [282] developed high-performance electrocatalysts using biomass-derived carbon supports (SLAC) from sugarcane leaves to enhance the performance of direct ethanol fuel cells (DEFCs). N-doped SLAC improves performance by providing uniform metal dispersion, thereby enhancing electron-rich sites. PtSnO_2_/N-doped SLAC exhibits outstanding performance, achieving the lowest onset potential, the highest current density, and enhanced mass activity during ethanol oxidation reaction (EOR) tests. This improvement is attributed to the synergistic effect of N-doped SLAC and SnO_2_, which provide electron-rich sites and improve CO tolerance by removing CO adsorption on the Pt surface via oxygen. Redox reactions remain the performance-limiting step in fuel cells due to slow kinetics and the high cost of platinum-based catalysts. Beygisangchin et al. [283] synthesized bio-based CDs from bagasse extract via a green route, which exhibited optimal electrocatalytic performance in the methanol oxidation reaction (MOR): their electrochemical active surface area reached 98.87 m^2^ g^−1^, mass activity was 688.25 mA mg^−1^, and they also possessed excellent anti-CO poisoning capability. Their strong stability was confirmed by amperometry and cyclic voltammetry tests, with a current retention rate of 45% after 1000 cycles. Arévalo-Cid et al. [284] synthesized nitrogen-doped carbon electrocatalysts using wet grape stalks without chemical pretreatment. Through a three-step process of hydrothermal carbonization, mechanochemical activation with dicyandiamide, and high-temperature pyrolysis, mesoporous carbon materials rich in pyridinic nitrogen and metal-nitrogen coordinated active sites were successfully constructed. Among them, Fe-based catalysts exhibited the optimal performance due to the synergistic effect of in situ-formed Fe_3_C active sites, nitrogen-doped carbon matrix, and high specific surface area—their oxygen reduction reaction (ORR) activity and stability were superior to the control system, electrochemical performance was comparable to commercial Pt/C catalysts, and they simultaneously possessed significantly reduced cost and environmental footprint. This material provides a high-performance platinum-group metal-free cathode catalyst solution for anion exchange membrane fuel cells (AEMFCs), while realizing the resource utilization of agricultural waste and promoting the decarbonization of hydrogen energy technology and the development of a circular economy.

## 6. Conclusions and Future Perspectives

This review presents a comprehensive review of the green synthesis technologies, machine learning-aided synthesis, and diversified application fields of CDs. Specifically, this review first outlines the research background and cutting-edge progress of fluorescent carbon dots and clarifies their research value and application potential against the backdrop of green, low-carbon, intelligent, and high-efficiency development. Subsequently, it systematically discusses the characteristics and applications of two categories of precursors. Natural precursors (e.g., plants, fruit peels) are in line with the concept of green synthesis owing to their wide availability, environmental friendliness, and low cost. On this basis, this review elaborates on two representative synthetic strategies of fluorescent carbon dots, namely top-down and bottom-up approaches, analyzes the advantages and applicable scenarios of each method, and provides technical support for the green, efficient, and large-scale preparation of such materials.

This review focuses on the innovative applications of machine learning in the synthesis of fluorescent carbon dots, and deeply investigates its core roles in the precise modulation of emission wavelengths, as well as the enhancement and prediction of fluorescence quantum yields. It validates that machine learning can overcome the limitations of conventional experiments and realize the intelligent optimization of synthetic procedures. Meanwhile, this review comprehensively summarizes the multifarious application scenarios of fluorescent carbon dots, including sensing (metal ion detection and food safety detection, where the integration of machine learning effectively improves detection accuracy), biomedicine (bioimaging and drug delivery), information anti-counterfeiting, luminescent devices, corrosion inhibition and protection, photocatalysis, and electrocatalysis. These applications fully demonstrate that CDs possess the core merits of environmental compatibility and multifunctionality, exhibiting promising prospects for industrial-scale applications. Furthermore, centered on the two innovative themes of “green synthesis” and “AI assistance”, this review integrates the key achievements across various research stages, laying a solid theoretical and practical foundation for subsequent investigations in this field.

Although remarkable advances have been achieved in the green synthesis and AI-assisted applications of fluorescent carbon dots, numerous urgent challenges remain in this domain, pointing to critical directions for future research. In terms of green synthesis, several natural precursors suffer from complex compositions and poor performance reproducibility, and some auxiliary synthetic reagents are yet to be fully environmentally benign. Therefore, the key bottlenecks to be addressed in future studies include screening natural precursors with high activity and stable composition, developing green synthetic protocols with low cost and zero secondary pollution, and realizing the large-scale green fabrication of fluorescent carbon dots. For machine learning-assisted applications, current studies are mostly confined to the modulation and prediction of emission wavelengths and fluorescence quantum yields, necessitating a broader expansion of application scopes. In addition, the training of machine learning models largely relies on limited experimental datasets, compromising the generalization ability of models. Meanwhile, the intrinsic correlation between machine learning and synthetic processes, as well as property regulation, has not been fully elucidated. Further in-depth exploration is thus required to achieve the deep integration of models and experiments, and to further leverage the core function of AI in process optimization and property prediction.

Inadequate product purity and underdeveloped characterization systems remain the most fundamental unresolved challenge in the CD field [285,286], affecting all stages from synthesis evaluation and ML modeling to application validation. Non-standard purification causes large variations in product composition, and the lack of unified purity standards and characterization methods prevents reliable differentiation between CDs and molecular impurities, as well as quantitative analysis of component contributions, making cross-study comparisons unreliable. Furthermore, low-quality heterogeneous data severely limits ML model performance and has become a key bottleneck for AI integration. Future research should prioritize this issue by developing pure-phase CD synthesis methods to establish robust structure–property relationships and enable precise property modulation.

In bio-oriented application research, the performance stability of fluorescent CDs in biological systems, their service life in vivo and biological environments, and the cost of large-scale application remain key factors limiting their industrial translation for biological uses. Different biological application scenarios (e.g., bioimaging, drug delivery, and biosensing) impose distinctly different requirements on the biological properties of fluorescent CDs, such as biocompatibility, biodegradability, and targeting ability, calling for targeted studies to precisely modulate their biological properties and enhance adaptability to specific biological scenarios. Moreover, exploration of the underlying mechanisms of fluorescent CDs remains inadequate, particularly for those synthesized via ML-guided strategies, where the structure–biological property relationship remains unclear. Thus, future research should prioritize addressing these challenges by further optimizing green synthesis processes to ensure CD biosafety and biocompatibility, expanding the scope and accuracy of ML in regulating CD biological properties, and exploring the structure–biological property relationship and their biological functional mechanisms. These efforts will advance fluorescent CDs toward greenization, intellectualization, large-scale production, and multifunctional integration, fully unlocking their application potential in biological and related fields.

## Figures and Tables

**Table 3:** Comparison of different methods for the synthesis of CDs.

Method Categories	Specific Methods	Advantages	Disadvantages
Top-down	Laser ablation	Simple operation steps, high product purity, and rapid optimization of particle size via regulating laser parameters	High equipment cost; extremely low raw material utilization rate; great difficulty in large-scale production
	Arc discharge	Fast reaction rate, enabling preparation of doped CDs	Difficult precise regulation of the reaction process, prone to generating by-products; high safety risks
	Electrochemical oxidation	Mild reaction conditions, precise control of product size via regulating voltage/current; simple equipment	Low reaction efficiency; products are prone to agglomeration; passivation layer is easy to form on the electrode surface
	Ultrasonic treatment	Low equipment cost; safe operation without high temperature and pressure	Low cutting efficiency of carbon materials; uneven product particle size and easy agglomeration
Bottom-up	Hydrothermal /solvothermal	Cheap and easily accessible raw materials; safe operation and easy large-scale production; controllable CDs functions via raw material design	Long reaction time; uneven particle size requiring purification; safety risks associated with high temperature and pressure
	Microwave	Extremely fast reaction rate; low energy consumption; high product purity	Local overheating prone to carbonization; high safety risks
	Combustion	Small particle size and high graphitization degree	Difficult control of the combustion process; products are prone to agglomeration
	Template synthesis	Excellent uniformity of morphology/size; customizable pore structure; excellent product dispersibility	High cost of templates; environmental pollution risk from acid-soluble templates; cumbersome operation steps

**Table 4:** Different ML models for the synthesis of CDs.

Type of ML	Model Input(s)	Model Output(s)	Performance	Ref.
Gradient boosting decision tree-based model (XGBoost)	Reaction temperature (T), reaction time (t), type of catalyst (C), volume/mass of catalyst (VC), type of solution (S), volume of solution (VS), ramp rate (Rr), and mass of precursor (Mp)	Full-color PL wavelength and high PLQY	MSE < 0.1	[167]
1D deep convolution neural network (DCNN)	Precursor, mass, temperature, solvent, and reaction time	Spectral properties and fluorescence color	R^2^ = 0.82	[155]
Pearson’s correlation analysis and gradient-boosting decision-tree regression model	The type of farm waste (class); cellulose, Hemicellulose, lignin, ash, moisture, N, C, and C/N contents of the samples; pyrolysis temperature T; and residence time.	PLQYs	R^2^ > 0.9, RMSE < 0.02, MAPE < 3	[168]
Artificial neural network; ordinary least-squares regression; ANOVA modules	Precursor molar ratios, method, solvent, purification method, pH, reaction temperature (K), and reaction time (min)	Fluorescence color and emission wavelengths	R^2^ = 0.94; MAE = 25.8 nm	[166]

**Table 5:** ML-assisted detection applications of CDs.

Sources	Applications	Accuracy (%)	Ref.
Microcrystalline cellulose extracted from biological waste	Detection of Zn^2+^	82.4	[199]
Farmland waste	Detection of sulfonamide antibiotics	98.96	[201]
Citric acid, urea, o-phenylenediamine	Pesticide classification and detection	99.3	[202]
Herb	Detection of warfarin and its metabolites	100	[200]
Citric acid, 2,5-diaminobenzotrifluoride	Antibiotic qualitative analysis	98.57	[206]
Cetylpyridinium chloride	Detection of heavy metal ions (Cr^6+^, Fe^3+^, Fe^2+^ and Hg^2+^)	95	[207]
Ink, dimer acid diisocyanate	Detection of amoxicillin	95	[16]
Phenylethanol, hydrophobic deep eutectic solvents	Detection of trace Fe^3+^ in serum	99.16	[208]
Citric acid, urea, triethyl hydrochloride	Simultaneous detection of cytosine and 5-methylcytosine	RSD < 5.9%	[17]
Peanut shell	Detection of antibiotics	98.21	[209]
Quinaldine red	Detection of heavy metal ions (Cr^6+^, Fe^2+^, Cu^2+^, Fe^3+^, Mn^2+^, Co^2+^ and Ni^2+^)	95.6	[210]
Citric acid, cysteamine hydrochloride	Detection of trace Hg^2+^ and S^2−^	RSD < 4.27%	[211]
O-phenylenediamine, urea and sulfuric acid	Detection of tetracycline antibiotics in food	100	[212]
Marigold extract	Fingerprint recognition	86.94	[213]
Ammonium dihydrogen citrate, polymyxin B, ampicillin and gentamicin	Bacterial identification and detection	100	[179]
O-phenylenediamine	Detection of metal ions	99.5	[214]
Ammonium citrate, ampicillin, polymyxin and gentamicin	Rapid identification of bacteria on pork	100	[215]
Citric acid, 1,2-diaminobenzene	Detection of Cr^6+^ in groundwater and drinking water	95.71, 96.81	[164]
Urea, diaminopyridine	Detection of oral bacteria	100	[216]

**Table 6:** Smartphone-based CD sensing.

Materials	Applications	Linear Range (μM)	LOD (μM)	Ref.
CDs	Detection of amoxicillin	5–1000	2.80	[16]
BU-CDs/GN-QDs/RD-QDs	Detection of Hg^2+^ and S^2−^ in water and seafood	1.50−128.00 5.00−580.00	0.834, 3.885	[211]
CDs	Detection of cytosine and 5-methylcytosine	200–1000	43.40, 74.40	[17]
D-CDs@Hg^2+^, D- CDs@Hg^2+^@GSH	Detection of glutathione	0–400	1.84	[217]
AIS QDs	Detection of chlortetracycline	0.05–5.00	1.97	[218]
BCDs/Ag^+^/OPD	Detection of glutathione and adenosine deaminase	0.1–200.0 0.5-160.0	0.07, 0.09	[219]
CQDs/Fe^3+^	Detection of trace F^−^ in water samples	0–1053	15.79	[163]
Y-CDs	Detection of bilirubin	4.0–225.0	1.37	[220]
NB-CQDs	Detection of dopamine	5–500	3.31	[221]
GS-CDs	Detection of uric acid in saliva	0–500	10.80	[18]
BY-CDs	Detection of food additive brilliant blue	0.01–120.00	0.0081	[222]
N, S-CDs	Detection of Mn^2+^ in cosmetics	1–5	0.5	[223]
B/R-CDs	Detection of methylene blue	0.05–10.00	15	[224]
N-CQDs	Detection of Fe^3+^ and ascorbic acid	2–150 30-130	0.253, 1.57	[225]
YG-CDs	Detection of thiophanate-methyl in agricultural products	0–10	0.0507	[226]
Au-NCs	Quantitative detection of captopril	0.25–50.00	0.1013	[227]
BOCDs/PHP	Detection of glutathione	0.01–8.0	0.00333	[228]
B, N-CDs	Detection of propyl gallate under irradiation of UV and sun lamp	0.5–60.0	0.42, 0.30	[229]
HAc-CDs	Detection of creatinine	3.0 × 10^−4^–1000	8.4 × 10^−5^	[230]
COFs-Pb^2+^	Dual-mode detection of Pb^2+^	1 × 10^−4^–1	5.0 × 10^−5^	[231]
OVA-Ce/Ni NCs	Detection of doxycycline	0.2–80.0	0.051	[232]
ZnO, N-GQDs@MIP	Determination of trace diuron	0.0214–0.2144	0.011	[233]
YCDs	Detection of Cr^6+^ in living cells	0–800	0.349	[234]
DE-CDs	Detection of ascorbic acid	0.1–100.0	0.0987	[235]
Ag NCs, Cu NCs	Visual detection of Cu^2+^	1.0–60.0	0.36	[236]
Cu NCs@PP	Detection of Congo red	0.5–160.0	0.085	[237]

## Data Availability

Not applicable.
